# The Impact of Young and/or Exercised Plasma Transfusions in Neurodegenerative Conditions: A Scoping Review

**DOI:** 10.14336/AD.2025.0849

**Published:** 2026-10-16

**Authors:** Michael J. Kirton, Emily R. Cox, Ulrik Wisløff, Atefe R. Tari, Lance C. Dalleck, Claire Drummond, Belinda Lange, Ranjay Chakraborty, Kym Williams, Norma Bulamu, Alline Beleigoli, Joyce S. Ramos

**Affiliations:** ^1^Flinders University, College of Nursing and Health Sciences, Caring Futures Institute, Adelaide, South Australia, Australia.; ^2^Centre for Active Living and Learning, School of Education, University of Newcastle, Callaghan, NSW, Australia.; ^3^Active Living and Learning Research Program, Hunter Medical Research Institute, New Lambton, New South Wales, Australia.; ^4^The Cardiac Exercise Research Group at the Faculty of Medicine and Health Sciences, Department of Circulation and Medical Imaging, Norwegian University of Science and Technology, Trondheim, Norway.; ^5^Department of Neurology and Clinical Neurophysiology, St Olavs University Hospital, Trondheim, Norway.; ^6^Western Colorado University, Recreation, Exercise, and Sport Science Department, Gunnison, CO 81231, USA.; ^7^Flinders University, College of Medicine and Public Health, Flinders Health and Medical Research Institute, Adelaide, South Australia, Australia.; ^8^Flinders University, College of Science and Engineering, Medical Device Research Institute, Adelaide, South Australia, Australia

**Keywords:** Plasma exchange, blood plasma, safety, neurodegenerative diseases

## Abstract

The objectives of this scoping review were to examine, synthesise and present the current research literature on the impacts of transfusions of plasma from young (YPTs) and/or exercised (EPTs) individuals in clinical studies and pre-clinical models of neurodegenerative conditions. We also identify gaps in the existing literature and pinpoint areas for future research. Research literature that investigated YPTs/EPTs in neurodegenerative conditions, written in English, and published between January 2004 and June 2024 were considered for inclusion. Published and unpublished articles were located through databases including PubMed Central, MEDLINE, Cochrane Database of Systematic Reviews, Scopus, ProQuest, SPORTDiscus, and PROSPERO. Studies that included adults or animal models with neurodegenerative conditions were considered. Nineteen eligible studies investigated either YPTs (n=13), EPTs (n=3), or both plasma types (n=3). The available evidence suggests that both YPTs and EPTs may have beneficial impacts on neurodegenerative conditions. While beneficial impacts on cognition (n=11), neuroinflammation (n=6), neurogenesis (n=5), apoptosis (n=2) and synaptic function/synaptic proteins (n=6) are commonly reported, findings across studies remain inconsistent. Differences in the length of intervention, dosages and plasma type may explain the variation in results. However, more research is needed to conclusively determine the specific impacts and mechanisms of YPTs and EPTs. All included studies that assessed safety (n=5), concluded YPTs and EPTs to be safe and well tolerated. Overall, the available evidence regarding the impacts of YPTs and EPTs on neurodegenerative conditions is promising. However, further investigation is needed to ascertain the exact impacts, safety, mechanisms, and feasibility of YPTs and EPTs in neurodegenerative conditions.

## INTRODUCTION

Neurodegenerative conditions refer to a group of conditions characterised by the progressive loss of neuronal functions [1]. Neurodegenerative conditions include Parkinson’s disease (PD), motor neuron disease (MND), Alzheimer’s disease (AD), multiple sclerosis (MS), and dementia-related disorders (collectively termed tauopathies) [2, 3]. A common feature among many neurodegenerative conditions is the aggregation of aberrantly polymerised proteins with altered physicochemical properties, commonly termed misfolded proteins [1, 3]. This misfolding results in the function of these proteins being altered [2]. Neurodegenerative conditions possess a unique pathophysiology and aetiology that is not clearly understood [1]. Thus, treatment of these conditions is challenging and typically focuses on reducing the impacts of impairments or management of symptoms, rather than on preventing disease progression [4]. Neurodegenerative conditions affect millions of people worldwide and are considered major contributors to the overall disease burden [3, 5]. With an ever growing and aging population, the prevalence of neurodegenerative conditions is expected to continue to rise [1].

Despite a limited understanding of the pathophysiology and aetiology, there is some evidence showing that the development of neurodegenerative conditions is a direct or indirect result of non-modifiable and modifiable risk factors [3, 6]. Aging, a non-modifiable risk factor, is the single greatest contributor to the development of neurodegenerative conditions [3]. Alternatively, modifiable risk factors include obesity, hypertension, physical inactivity, and diabetes [7]. Exercise has been shown to have significant favourable effects on all the aforementioned modifiable risk factors [6, 8-10]. Furthermore, exercise is a well-established method of positively influencing brain cognition, neurogenesis and plasticity [10]. However, there are significant barriers to exercise in neurodegenerative populations that lead to poor long term exercise adherence [11-13]. Barriers include functional or physiological impairment, fear of falling, lack of time, and low outcome expectation [11-13]. Therefore, alternative/adjunct therapies are warranted that may overcome exercise barriers in this population, whilst still providing similar benefits.

Blood plasma transfusions is an emerging potential therapy that may overcome some of the exercise barriers observed in people with neurodegenerative conditions [6]. Evidence suggests that plasma transfusions from exercise trained individuals (termed exercised plasma, EPTs) may confer the beneficial effects of exercise and help to alleviate symptoms, and potentially the progression, of some neurodegenerative conditions [6,14-17]. Specifically, there are multiple studies in animal models that show the beneficial impacts of EPTs [14-17]. One trial [14] found EPTs from aged, exercised donor mice conferred the beneficial impacts of exercise on cognition and hippocampal neurogenesis in aged sedentary mice. In humans, one study [6] is currently in progress which is investigating the safety and efficacy of EPTs in people with AD and is expected to be completed in 2026.

Young plasma transfusions (YPTs) are another type of plasma transfusion that warrants investigation. A larger body of evidence suggests that transfusion of plasma from younger individuals (termed young plasma) may also have rejuvenating properties that alleviates the symptoms and progression of neurodegenerative conditions [18-21]. In mice models, YPTs has been demonstrated to reverse some of the various age-related changes in the mouse brain including, but not limited to, neuron degeneration and cognitive impairment [22]. Similar findings have been reported in clinical human studies using YPTs [23, 24]. Specifically in humans, platelet rich plasma infusions have been shown to reduce neuroinflammatory compounds including tumor necrosis factor-α (TNF-α) which has a systemic anti-inflammatory effect [23]. Furthermore, YPTs have been shown to be safe and well tolerated in both humans and animals, with no major adverse events or reactions reported [18, 23-25]. These may be important findings given the age-related risk factor in the development of neurodegenerative conditions [19].

### Rationale for the scoping review

To scope the current research on the impacts of YPTs and EPTs in clinical studies and pre-clinical models of neurodegenerative conditions, a preliminary search of PubMed, the Cochrane Database of Systematic Reviews, Google scholar, JBI Evidence Synthesis, and PROSPERO was conducted, and no current or underway systematic reviews or scoping reviews were identified. However, we identified several peer reviewed original trials on the safety, tolerability, and/or impact of plasma transfusions (young or exercised) in various neurodegenerative conditions [14, 23, 24]. Our preliminary search also identified one systematic scoping review which investigated the safety and efficacy of replacement fluids in therapeutic plasma exchange [26]. However, the impacts of YPTs and EPTs in neurodegenerative conditions were not explored in that study. The objective of this scoping review was to examine, synthesise, and present the current body of research on the impacts and application of plasma transfusions - whether from young or exercised donors - in individuals or animal models with neurodegenerative conditions, as well as to identify knowledge gaps and directions for future research.

### Review questions

The primary research question of this scoping review was:

- What are the impacts of plasma transfusions (young or exercised) in people or animal models with neurodegenerative conditions^+^
  The secondary research questions of this scoping review were:
- What is known about the safety and tolerability of YPTs and EPTs in people and animal models with neurodegenerative conditions, and what physiological parameters are measured to monitor the safety and tolerability of these therapies^+^
- What are the protocols implementing YPTs and EPTs in people or animal models with neurodegenerative conditions (intervention period, dosage, and number of transfusions)^+^
- What is the cost effectiveness of YPTs and EPTs in people with neurodegenerative conditions^+^
- What are the barriers and facilitators to effective implementation of plasma transfusions (young or exercised) in people and animal models with neurodegenerative conditions^+^

## METHODS

This review was conducted in accordance with an a-priori protocol [27] and with the JBI methodology for scoping reviews and the Preferred Reporting Items for Systematic Reviews and Meta Analyses Extension for Scoping Reviews (PRISMA-Scr) (Appendix I) [28, 29].

### Eligibility criteria

#### Participants

Adult human participants and animal models who had one of the following neurodegenerative conditions were included: AD, PD, Huntington’s disease, MND, amyotrophic lateral sclerosis, vascular dementia, Lewy body dementia, spinocerebellar ataxia, MS, and prion disease. Additionally, older adults and models of aged animals were included due to the age-related risk factor in many neurodegenerative conditions [19].

#### Concepts

The key concepts addressed were YPTs and EPTs. Studies were excluded if they focused on the impacts of other types of plasma or replacement fluids that were not young or exercised plasma, or on the application of YPTs and EPTs in non-neurodegenerative conditions.

#### Plasma transfusion and exchange

Plasma transfusion is the process of administering donor plasma into a patient’s blood stream. Plasma exchange involves blood being removed from an individual (patient) using an intravenous needle into an exchange machine. This machine separates the different components of blood (e.g. plasma), after which the new plasma (young or exercised) is added and returned to the patient.

#### Young plasma

Young animal donors (typically mice) are usually aged 3-4 months old [25], whereas young human donors are typically aged between 18-30 years old [24].

#### Exercised plasma

Plasma drawn from exercise trained donors is considered exercised plasma. Currently there is not a clearly defined donor criteria for exercised plasma donors, as this is a relatively new experimental therapy. Of the available research protocols, male donors were aged 18-40 years old, had a body mass index of ≤27 kg/m^2^, and a maximal oxygen uptake (VO^2max^) of ≥55 mL/kg/min [6]. Animal (mice) donor criteria included four-month-old mice that had undergone high-intensity interval training five days per week for six weeks [15, 17].

#### Context

The global published and unpublished research on the impacts of YPTs and EPTs in people and in animal models with neurodegenerative conditions.

#### Information sources

This scoping review included both experimental and quasi-experimental study designs, encompassing randomised controlled trials, controlled clinical trials, non-randomised controlled trials, as well as pre-post intervention studies, with or without control groups. To maximise available information on protocol development, protocol papers were also included. Only studies published in English were considered. Eligible studies were those published between January 2004 and June 21, 2024, to capture the contemporary use of plasma transfusions. Systematic reviews meeting the inclusion criteria were considered when relevant to the research question. Additionally, qualitative studies were included if they provided relevant qualitative data, encompassing methodologies such as phenomenology, grounded theory, ethnography, qualitative description, and action research.

#### Search strategy

The search strategy aimed to locate both published and unpublished studies. A three-step search strategy was utilized. First, an initial limited search of PubMed and Google scholar using keywords was undertaken to identify articles on the topic (Appendix II, Supplementary Table 1). The text words contained in the titles and abstracts of relevant articles, and the index terms were used to develop the search strategy. The full search strategy is provided in Appendix II and was used to scope the following sources of research:

Electronic databases: PubMed Central, SPORTDiscus, Scopus, MEDLINE, ProQuest, and Cochrane Database of Systematic Reviews.

Trial registries: PROSPERO

Unpublished studies: The search for unpublished studies included theses, book chapters, and conference papers.

The search strategy, including all identified keywords and index terms, were adapted for each included database and/or information source. The reference lists of all included sources of evidence were screened for additional eligible studies.

#### Study selection

Following the search, all identified citations were uploaded into Endnote V21.2/2024 (Clarivate Analytics, PA, USA) and duplicates removed. Relevant sources were retrieved in full, and their citation details imported into the Covidence systematic review software, Veritas Health Innovation (Covidence, Melbourne, Australia). Titles and abstracts were then screened by two independent reviewers (MK and ERC) for assessment against the inclusion criteria for the review.

The full text of selected citations was assessed in detail against the inclusion criteria by two independent reviewers (MK and ERC). Any difference of opinion between the reviewers were resolved through discussion, or with an additional reviewer (JSR).

#### Data extraction

Data was extracted from papers included in the scoping review by two independent reviewers (MK and ERC) using a modified JBI data extraction tool and a modified preliminary extraction tool (Appendix III, Supplementary Table 2). The data extracted included specific details about the authors; year and country of publication; aims, purpose or objectives of the study; sample size; participants; condition/s; age; type of plasma used, protocols for plasma transfusion; and key findings related to the scoping review questions. Any disagreements that arose between the reviewers were resolved through discussion, or with an additional reviewer (JSR). When required, authors of papers were contacted to request missing or additional data.

#### Data Analysis and Presentation

Key findings of included studies were presented in tabular form with appropriate narrative synthesis. The method of data analysis and presentation was chosen because of the heterogeneity of included participants and studies, to allow for multiple types of transfusion and neurodegenerative conditions to be included, and the relatively new nature of the field. Additionally, a PRISMA flow diagram (Appendix IV, Supplementary Fig. 1) was modified to report the results of the literature search [29].

#### Quality assessment of included studies

The methodological quality of the included animal model studies were assessed using the Systematic Review Centre for Laboratory animal Experimentation (SYRCLE) risk of bias tool [30] (Appendix V, Supplementary Table 3 and Supplementary Figure 2). The answer spectrum indicated “No” for a high risk of bias, “Unclear” for an unclear risk of bias, and “Yes” for a low risk of bias. The included clinical studies were assessed for methodological quality using the Physiotherapy Evidence Database (PEDro) scale criteria [31] (Appendix V, Supplementary Table 4). Studies with a PEDro score of ≤3 were classified as poor, fair for 4-5, and good for a score ≥6 [31].

### RESULTS

#### Study inclusion

From the literature search conducted 3,646 citations and 13 grey literature articles were identified, from which 1,432 duplicates were removed. The titles and abstracts of the remaining 2,227 records were screened for eligibility. After screening, 1,992 records did not meet the eligibility criteria. The full texts of 235 papers were reviewed to further assess eligibility, of which 219 were excluded with justification (Appendix IV, Supplementary Figure 1). Additionally, a further three studies were found in the citations of the full text review papers and included in the review. Therefore, in total, the final data set included 19 studies. The search results are summarised in the PRISMA flow diagram (Appendix IV, Supplementary Fig. 1).

#### Characteristics of included studies

Six of the included studies were published prior to 2020 [21, 24, 32-35], five studies were published in 2020 [14, 16, 23, 25, 36], two in 2021 [18, 37], one in 2022 [6], three in 2023 [19, 20, 38] and two studies were published in 2024 [15, 17]. Thirteen studies were from the United states of America [14, 19-21, 23-25, 32, 34-38], three from Norway [6, 15, 17], and the remaining three studies were from South Korea [16], China [33], and Iran [18]. Of the 19 studies reviewed, 18 studies reported a sample size of between 3-18 participants that received either YPTs or EPTs (Table 1). The remaining included study [6] was a protocol paper which aims to recruit 20 participants.

**Table 1:** Characteristics and key findings of included studies.

Study#	Authors	Participant and Donor Characteristics	Plasma type and Protocols	Key Findings			Compared to:
				YPT over Controls	EPT over Controls	EPT over YPT	
**Human Studies**							
**1**	Parker et al. [23], USA	Participants: 15 people with PD with at least 1 cognitive complaint, and on stable therapy, age: 68 ± 8.3, male (n = 10) female (n = 5) Donors: Young fresh frozen plasma, other donor characteristics not specified	Young, 28 days, 8 doses, 250mL per dose	↔ Cognition (↔ TMT, ↑ Phonemic fluency), ↔ QOL (↔ Blinded UPDRS III, ↓ PDQ39 stigma sub score, ↔ PDQ39 total score, ↓ Unblinded UPDRS III), ↔ Psychiatric symptoms/mood (↔ BDI, BAI)			Baseline measures
**2**	Sha et al. [24], USA	Participants: 18 people with AD, age: 74.17 ±7.96 years, 12 women (n = 12) and men (n = 6) Donors: Young fresh frozen plasma (male only, age 18-30 years)	Young, 28 days, 4 doses, 250mL per dose	↔ Cognition (↔ ADAS-Cog 13, TMT part A, CDRSSB), ↔ Psychiatric symptoms/mood (↔ GDS, NI), ↑ QOL (↑ ADCS-MCI-ADL, ↓ FAQ)			Placebo
**3**	Tari et al. [6], Norway	Participants: 20 people with AD aged up to 75 years, with a diagnosis of AD in early phase Donors: Adults, age: 18-40 years, BMI ≤27 kg/m^2^ and maximal oxygen uptake >55 mL/kg/min, male	Exercised, 1 year (3x 4 week blocks), 12 doses, 200 mL per dose	No key findings, protocol only paper			Regular plasma (octa-plasma) and saline
**Animal Model Studies**							
**4**	Castellano et al. [32], USA	Participants: 6 aged NSG Mice, age: 13.8±0.2 months old, male and female Donors: Human, elderly age: 61-82yrs, young age: 19-24yrs, cord from neonates (at birth)	Young, 14-21 days, 6-8 doses, 175μl per dose	↑ Cognition (↑ CFOS, EGR1)			Vehicle plasma
**5**	Hahn et al. [19], USA	Participants: 3-4 aged C57BL/6JN strain mice, age: 19 months, male Donors: C57Bl/6J mice, age: 2 months, male	Young, 28 days, 7-9 doses, 150 μl per dose	↑ Neurogenesis (↑ Neurogenic lineage and ↓ age-related DEGs found in aNSCs)			Saline
**6**	Hernandez et al. [20], USA	Participants: 15 aged AD mice models (rTg4510 mice expressing human tau), age: 8 months, male and female Donors: Young wildtype mice, age: 2-3 months, male and female	Young, 16 days, 8 doses, 150 µL per dose	↔ Cognition (↔ Nesting, burrowing, OFT, MWM test), ↓ Phosphorylated Tau, ↓ Tau tangles			Saline
**7**	Schroer et al. [38], USA	Participants: 6-15 aged mice, age: 20 months, male Donors: Mice, age: 3 months, male	Young, 24 days, 8 doses, 100 μl per dose	↓ Neuroinflammation (↓ TNF, C1qb, CD11b, IBA1-positive microglia, CD68+)			Saline
**8**	Smith et al. [36], USA	Participants: 4-8 aged mice, age: 17-18 months, male Donors: Mice, age: 2-3 months, male	Young, 24 days, 8 doses, 100 ul per dose	↑ Cognition (↑ FCP), ↑ Neurogenesis (↑ DCX+), ↑ Synaptic marker expression (↑ pCREB, BDNF, Synapsin-1, PSD95)			Saline
**9**	Xia et al. [33], China	Participants: 8 AD mice models (APP/PS1 mice), age: 12 months, male Donors: Mice, age: 3 months, male	Young, 30 days, 10 doses, 100 μl per dose	↑ Cognition (↑ Y-maze, NORT, OFT), ↓ Aβ Pathology, ↑ Synaptic pathology (↑ Synaptogenesis, Synaptic plasticity, Synaptophysin, REST/FOXO1 signalling, pCREB, BDNF), ↑ Psychiatric symptoms/mood (↑ OFT)			Aged plasma
**10**	Zhao et al. [25], USA	Participants: 10 AD mice models (3×Tg-AD mice), age: 16-17 months, female Donors: C57BL/6 J wild-type mice, age: 2-3 months, male and female	Young, 56 days, 16 doses, 150 μl per dose	↑ Cognition (↑ NORT, ↓ MWM, reversal MWM), ↓ Neuroinflammation (↓ IBA 1), ↓ Tau, ↓ Aβ, ↔ Neurogenesis (↔ NeuN), ↔ Synaptic proteins (↔ pCREB, synapsin 1, synaptophysin, PSD95)			Saline
**11**	Villeda et al. [34], USA	Participants: 8 aged C57BL/6 mice, age: 18 months, male Donors: Mice, age: 3 months, male	Young, 24 days, 8 doses, 100 μl per dose	↑ Cognition (↑ FCP, RAWM), ↑ Synaptic proteins (pCREB)			Aged plasma
**12**	Khrimian et al. [35], USA	Participants: 11-16 aged wild type mice, age: 16 months, female Donors: Wild type mice, age: 3 months, female	Young, 24 days, 8 doses, 100 μl per dose	↑ Cognition (↑ NORT), ↓ Anxiety like behaviours (↓ EPMT)			Aged plasma
**13**	Ghaffari-Nasab et al. [18], Iran	Participants: 8 aged (depression model) rats, age: 22 months, male Donors: Rats, age: 3 months, male	Young, 28 days, 12 doses, 1ml per dose	↓ CUMs induced depression like behaviours (↑ sucrose preference test, ↑ OFT), ↓Neuronal apoptosis (↓ TUNEL-positive cells, CC3)			Aged CUMs (depressed) rats
**14**	Middeldorp et al. [21], USA	Participants: 13 AD mice models (APP mice), age: 10-12 months, female Donors: Mice, age: 2-3 months, female	Young, 28 days, 8 doses, 150 µL per dose	↑ Cognition (↑ Y-maze, FCP), ↑ Synaptic proteins (↑ Synaptophysin, ERK), ↔ Aβ			PBS (saline)
**15**	DeMiguel et al. [37], USA	Participants: 8 LPS induced neuroinflammation mice, age: 3-4 months, male Donors: Mice given access to running wheel for 28 days, age: 3-4 months, male	Exercised, 6 hours, 3 doses, 200µl per dose		↑ Cognition (↑ MWM, FCP), ↑ Neurogenesis (↑ DCX+, BrdU/NeuN), ↓ Neuroinflammation (↓ Myd88, Stat1, Irak3)		Control plasma
**16**	Horowitz et al. [14], USA	Participants: 6-19 aged sedentary mice, age: 18 months, male Donors: Aged donor mice given continuous access to a running wheel in their cage for 42 days, age: 18 months, male; AND mature donor mice (same exercise protocol), age: 6-7 months, male	Exercised, 24 days, 8 doses, 100 μl per dose		↑ Cognition (↑ FCP, RAWM), ↑ Neurogenesis (↑ DCX+, BrdU/NeuN+), ↑ Synaptic proteins (↑ BDNF)		Sedentary control plasma
**17**	Kim et al. [16], South Korea	Participants: 10 AD mice models (3xTg-AD mice), age: 12 months, male Donors: Young mice, age: 4 months, male OR young exercised mice (training period - 5 times per week for 12 weeks), age: 3 months, male	Both Young and Young + Exercised, 30 days, 10 doses, 100-µL per dose	↔ Cognition (↔ MWM, STAT), ↑ Synaptic proteins (↑ BDNF, ↔ PSD95, Synaptophysin), ↔ Suppression of apoptosis (↔ Bax, Cytochrome c, Apaf-1, CC3 and 9, Bcl-2), ↔ Mitochondrial function, ↔ Mitochondrial Ca2+ retention capacity, ↔ H2O2 emission rate	↑ Cognition (↑ MWM, STAT), ↑ Neurogenesis (↑DCX+, BrdU/NeuN), ↑ Synaptic proteins (↑ BDNF, Synaptophysin, PSD95), ↓ Apoptosis (↓ Bax, Cytochrome c, Apaf-1, CC3 and 9, Bcl-2), ↔ Tau expression, ↑ Mitochondrial function, ↑ Mitochondrial Ca2+ retention capacity, ↓ H2O2 emission rate	↑ Cognition (↑ MWM, STAT), ↑ Neurogenesis (↑ DCX+, BrdU/NeuN), ↓ Apoptosis (↓ Bax, Cytochrome c, Apaf-1, CC3 and 9, Bcl-2), ↑ Synaptic proteins (↑BDNF, Synaptophysin, PSD95), ↔ Tau expression, ↑ Mitochondrial function, ↑ Mitochondrial Ca2+ retention capacity, ↓ H2O2 emission rate	Young plasma and a control group (AD mice model)
**18**	Norevik et al. [17], Norway	Participants: 7 (early stage AD model) and 7-9 (later stage AD model) transgenic rats (McGill-R-Thy1-APP), early pre-plaque pathology stage, age: 2.2 ±0.14 months OR later stage closer to the time point when extracellular plaques emerge, age: 5 ±0.20 months, male Donors: Exercise-trained young male Wistar rats (exercise training 5 days/week for 6 weeks), age: 1-2 months, male OR sedentary Wistar rats, age: 1-2 months, male	Both Young and Young + Exercised, 42 days, 14 doses, 2 mL/kg body weigh per dose	↔ Cognition (↔ FCP, NORT), ↔ Aβ	↔ Cognition (↔ FCP, NORT), ↑ Neurogenesis (↑ BrdU+, NeuN+), ↔ Aβ	↔ Cognition (↔ FCP, NORT), ↓ Neuroinflammation (↓ GM-CSF, GRO/KC, IL-1b, IL-7, MCP1, MIP-1a, MIP3a), ↔ Neurogenesis (↔ BrdU+, NeuN+), ↔ Synaptic proteins (↔ BDNF), ↔ Aβ	Young sedentary plasma and saline
**19**	Huuha et al. [15], Norway	Participants: 7 (early stage AD model) and 7 (later stage AD model) transgenic rats (McGill-R-Thy1-APP), early pre-plaque pathology stage, age: 2.2 ±0.14 months OR later stage closer to the time point when extracellular plaques emerge, age: 5 ±0.20 months, male Donors: Exercise-trained young male Wistar rats (exercise training 5 days/week for 6 weeks), age: 1-2 months, male OR sedentary Wistar rats, age: 1-2 months, male	Both Young and Young + Exercised, 42 days, 14 doses, 2 mL/kg body weigh per dose	↔ Cognition (↔NORT), ↑ Neuroinflammation (↑ TNF-a), ↔ Aβ, ↑ Microglial morphology	↔ Cognition (↔NORT), ↔ Neuroinflammation (↔ TNF-a), ↑ Aβ, ↑ Microglial morphology	↔ Cognition (↔NORT), ↓ Neuroinflammation (↓ TNF-a), ↔ Aβ, ↔ Microglial morphology	Young sedentary plasma and saline

↑ or ↓ indicates a significant beneficial impact between groups was reported relative to comparative group/baseline measure, ↔ indicates no significant difference was reported between groups. Abbreviations: Young plasma transfusion (YPT), Exercised plasma transfusion (EPT) Parkinson’s disease (PD), Trail Making Test (TMT), Quality of Life (QOL), Movement Disorder Society -Unified Parkinson’s Disease Rating Scale (UPDRS III), Parkinson's Disease Questionnaire - 39 (PDQ-39), Beck Depression Inventory (BDI), Beck Anxiety Inventory (BAI), Alzheimer’s disease (AD), Alzheimer Disease Assessment Scale-Cog (ADAS-Cog 13) 13-item version, Clinical Dementia Rating Scale Sum of Boxes (CDRSSB), Geriatric Depression Scale (GDS), Neuropsychiatric Inventory (NI), Alzheimer Disease Cooperative Study Activities of Daily Living Inventory in mild cognitive impairment (ADCS-MCI-ADL), Functional Activities Questionnaire (FAQ), Early Growth Response 1 (EGR1), Differentially expressed genes (DEGs), activated neural stem cells (aNSCs), open field test (OFT), Morris water maze (MWM), tumor necrosis factor (TNF), complement initiator C1qb (C1qb), microglia activation marker CD11b (CD11b), fear conditioning paradigm (FCP), doublecortin positive immature neurons (DCX+), phosphorylated cAMP response element-binding protein (pCREB), brain derived neurotrophic factor (BDNF), Postsynaptic density protein 95 (PD95), Y maze spontaneous test (Y-maze), novel object recognition test (NORT), β-amyloid peptide (Aβ), repressor element 1-silencing transcription factor (REST), Forkhead box protein O1 (FOXO1), hexaribonucleotide binding protein-3 (NeuN), radial arm water maze (RAWM), elevated plus maze test (EPMT), chronic unpredictable mild stress (CUMs), terminal deoxynucleotidyl transferase dUTP nick-end labelling (TUNEL), cleaved caspase-3/procaspase-3 ratio (CC3), extracellular receptor kinase (ERK), lipopolysaccharide (LPS), 5-bromo-2-deoxyuridine (BrdU+), step-through avoidance tasks (STAT), granulocyte macrophage colony stimulating factor (GM-CSF), growth-regulated oncogene/keratinocyte chemoattractant (GRO/KC), interleukin-1b (IL-1b), interleukin-7 (IL-7), monocyte chemoattractant protein-1 (MCP1), macrophage inflammatory protein-1a (MIP-1a), macrophage inflammatory protein-3a (MIP3a).

YPTs were the most frequently investigated plasma transfusion type (n= 13) [18-21, 23-25, 32-36, 38]. Three studies investigated [14, 37] or will investigate [6] EPTs. Lastly, three studies investigated both YPTs and EPTs [15-17]. Sixteen studies [14-21, 25, 32-38] used animal models to investigate plasma transfusions, thirteen studies used mice [14, 16, 19-21, 25, 32-38], and three studies used rats [15, 17, 18]. The remaining three studies focused on human participants [6, 23, 24]. Nine studies investigated plasma transfusions in people with AD or AD animal models [6, 15-17, 20, 21, 24, 25, 33]. Eight studies used aged animal models [14, 18, 19, 32, 34-36, 38]. De Miguel and collaborators [37] used lipopolysaccharide (LPS) mice models, which are commonly used to study the mechanisms of inflammation and neurodegeneration. Lastly, Parker et al. [23] investigated plasma transfusions in people with PD. A detailed summary of the study characteristics is reported in Table 1.

#### Assessment of study methodology quality

The included clinical studies had a PEDro score of 1/10 [23] and 5/10 [24]. The overall quality of the included clinical studies was considered to be poor [23] and fair [24]. None of the included clinical studies reported concealed allocation, assessor/subject/therapist blinding, point and variability measures or conducted an intention to treat analysis. Of the 16 animal model studies included in this review more than half did not report allocation concealment, random sequence generation, random housing, blinding (performance bias), and random outcome assessment.

##### Review Findings

#### Review question 1

What are the impacts of plasma transfusions (young or exercised) in people or animal models with neurodegenerative conditions^+^

Eighteen studies reported positive impacts of plasma transfusions (Table 1 and Table 2) [14-21, 23-25, 32-38]. The remaining study is a protocol paper [6] with the results yet to be published. Thirteen YPT studies [18-21, 23-25, 32-36, 38] reported positive impacts in neurodegenerative conditions or aged participants. Five studies reported positive impacts of EPTs [14-17, 37] with three of these studies investigating both EPTs and YPTs [15-17].

#### Cognition

Seven animal model studies [21, 25, 32-36] showed significant beneficial impacts on cognition or cognitive impairments, via improvements in memory and/or learning, after administration of YPTs. One study used quantitative polymerase chain reaction (qPCR) to analyse gene expression and found a significant increase in cFos and Early Growth Response 1 (EGR1) after YPTs compared to with controls (vehicle treated) [32]. This suggests an improvement in long term potentiation (LTP) and therefore an improvement in cognitive function [32]. The other animal model studies assessed cognition using various behavioural tests including the Y maze spontaneous test [21, 33], novel location recognition task [33], open field test (OFT)[20, 33], novel object recognition test (NORT) [15, 25, 35], Morris water maze (MWM) [16, 20, 25], reversal MWM [25], burrowing test [20], nesting test [20], fear conditioning paradigm (FCP) [21, 34, 36], step-through avoidance tasks [16], and radial arm water maze (RAWM)[34]. Three animal model studies [15, 16, 20] found no significant impacts on memory/behaviour (cognition) between aged/AD mice administered YPT and controls (no intervention). In human trials cognition was assessed via the trail making test (TMT)[23, 24], Alzheimer Disease Assessment Scale-Cog (ADAS-Cog 13) 13-item version [24], and the Clinical Dementia Rating Scale Sum of Boxes (CDRSSB) [24], with no significant changes reported in these assessments. However, Parker et al. [23] did report significant improvements in phonemic fluency after YPTs.

**Table 2:** Key findings of included articles presented as a percentage of included studies that showed significant beneficial impacts of plasma transfusions (young or exercised).

	Young plasma over control	Exercised plasma over control	Exercised plasma over young plasma
**Cognition**	58% [21, 25, 32-36]	60% [14, 16, 37]	33% [16]
**Neuroinflammation**	66% [25, 38]	50% [37]	100% [15, 17]
**Neurogenesis**	66% [19, 36]	100% [14, 16, 17, 37]	50% [16]
**Apoptosis**	50% [18]	100% [16]	100% [16]
Synapses/synaptic proteins*	83% [16, 21, 33, 34, 36]	100% [14, 16]	50% [16]
**Aβ**	40% [25, 33]	50% [15]	0%
**Tau**	100% [20, 25]	0%	0%
**Psychiatric symptoms/Mood**	60% [18, 33, 35]	No studies	No studies
**Quality of Life**	50% [24]	No studies	No studies

* At least one synaptic protein found to be significantly changed

Similar beneficial impacts were also shown in studies using EPTs [14, 16, 37]. Three studies [14, 16, 37] showed significant, positive impacts of EPTs on cognition and memory, via improvement in FCP [14, 37], MWM [16, 37], RAWM [14], or step through avoidance test [16], compared with controls (control plasma) [14, 37], and no intervention [16]. Furthermore, Kim et al. [16] found cognition and memory were also significantly improved following EPTs, compared with AD mice administered YPTs. Horowitz et al. [14] found a link between increased glycosylphosphatidylinositol-specific phospholipase D1 (Gpld1) concentrations in exercised mice plasma compared with age-matched sedentary mice plasma, as well as improvement in cognitive performance, via improvements in FCP and RAWM. Thus, the additional benefits observed after EPTs may be mediated by the increased concentrations of Gpld1 in exercised plasma [14]. However, two studies [15, 17] found no significant differences in cognitive function, via NORT [15, 17] and FCP [17], between EPTs, YPTs, or saline-treated rats.

#### Psychiatric Symptoms and Mood

In animal model studies, Ghaffari-Nasab et al. [18] found that YPTs were able to significantly reduce chronic unpredictable mild stress induced depression-like behaviours, via changes in sucrose preference test and OFT in aged rats. Khrimian et al. [35] found a significant reduction in anxiety like behaviours after YPTs, assessed via changes in elevated plus maze test score. Xia et al. [33] found a significant improvement in psychiatric symptoms via changes in OFT. Conversely no significant differences in psychiatric symptoms or mood measures were found in human trials after YPTs compared to controls (no intervention) [24] or baseline measures [23]. In human trials, the Beck anxiety and depression inventory scales [23], neuropsychiatric inventory [24], and the geriatric depression scale [24] were used to assess psychiatric symptoms and/or mood.

#### Neuroinflammation

Two [25, 38] animal model studies reported significant positive impacts of YPTs on neuroinflammation via a reduction in proinflammatory cytokines [25, 38] and markers [25]. With positive impacts on neuroinflammation also reported in people with PD, via a reduction in TNF-α, however statistical analysis was not completed for this measure [23]. Huuha et al. [15] reported a significant increase in neuroinflammation (increased TNF-α) after administration of young sedentary plasma to early-stage AD mice models. However, Huuha et al. [15] reported a significant reduction in neuroinflammation (TNF-α) after EPTs compared to young sedentary plasma, however no significant decrease was reported when comparing EPTs to controls (saline). Two other EPTs studies [17, 37] reported on neuroinflammation. De Miguel et al. [37] found EPTs significantly reduced neuroinflammation (via reduction in proinflammatory cytokines) compared with controls (no intervention). Norevik et al. [17] found significant reductions in neuroinflammation, via reduction in proinflammatory cytokines, when comparing EPTs with YPTs. Several of these studies [15, 23, 38] reported that the reduction in neuroinflammation was likely mediated through changes in TNF/TNF-α.

#### Amyloid β-protein and Tau

Four studies [15, 20, 25, 33] using AD animal models showed positive impacts on two key AD proteins tau tangles and amyloid β-protein (Aβ), which are closely linked to neuroinflammation and inflammatory processes, after YPTs and/or EPTs. Of these studies, two showed positive impacts on tau tangles [20, 25], and three showed positive impacts on Aβ pathology [15, 25, 33]. Kim et al. [16] found no significant changes in tau protein levels between mice administered EPTs or YPTs compared with controls (no intervention). Middeldorp et al. [21] and Norevik et al. [17] showed no significant reductions in Aβ burden in AD animal models after YPTs and/or EPTs.

Huuha et al. [15] reported EPTs modulated microglial morphology promoting microglial responses to early accumulation of Aβ. Specifically, they found the greater total branch length and number of branches for the microglia may suggest treatment impacts on intraneuronal Aβ and microglial response [15].

#### Synaptic function and protein expression

Five studies [16, 21, 33, 34, 36] showed positive impacts of YPTs on either synaptic marker expression [36], synaptogenesis [33], synaptic plasticity [33], and/or synaptic proteins [16, 21, 33, 34, 36]. Specifically, significant correlations were found between YPTs and commonly affected synaptic proteins in neuro-degenerative conditions including Brain-Derived Neurotrophic Factor (BDNF) [16, 33, 36], synaptophysin [21, 33, 36], Synapsin-1 [36], cAMP response element-binding protein (CREB) [33, 34, 36], anti-extracellular receptor kinase (ERK) [21], and postsynaptic density protein 95 (PSD95) [36]. However, Zhao et al. [25] found no significant difference after YPTs in synaptophysin, Synapsin-1, CREB, and PSD95, compared with controls (no intervention). Similarly Kim et al. [16] found no significant difference between YPTs and controls (no intervention) in synaptophysin and PSD95. Three studies [14, 16, 17] investigated EPTs and synaptic proteins. Horowitz et al. [14] found a significant increase in BDNF after EPTs compared to sedentary plasma transfusions. Kim et al. [16] found significant increases in BDNF, synaptophysin and PSD95 in the EPTs group compared with both the YPTs group and the control group (no intervention). Norevik et al. [17] found no significant difference in BDNF levels between EPTs or YPTs, though, BDNF was not assessed in the control group (no intervention).

#### Neurogenesis and apoptosis

Smith et al. [36] reported that YPTs promote neurogenesis in aged mice, via a significant increase in number of doublecortin positive immature neurons (DCX+), compared with a control group (no intervention). Hahn et al. [19] found YPTs downregulated age-related differentially expressed genes (DEGs) in activated neural stem cells (aNSCs) of aged mice. Concluding that YPTs reversed aging signatures of the neurogenic linage, indicating promotion of neurogenesis [19]. Zhao et al. [25] found no significant differences in neurogenesis, via number of NeuN markers, between AD mice models injected with young plasma or saline. Conversely, all four [14,16,17,37] EPTs studies that investigated neurogenesis found significant improvements in neurogenesis compared with controls (sedentary plasma [14, 37] or no intervention [16, 17]). De Miguel et al. [37] found an increase in the total number of proliferating cells, DCX+ neuroblasts and surviving cells after EPTs compared to control plasma (non-exercised) mice. Horowitz et al. [14] found an increase in total number of DCX+, 5-bromo-2-deoxyuridine (BrdU+), and hexari-bonucleotide binding protein-3 (NeuN+) in the dentate gyrus of the hippocampus after EPTs. Norevik et al. [17] showed an increase in BrdU+ and NeuN+ cells following EPTs. Kim et al. [16] found increased levels of DCX+, BrdU+, NeuN+ cells after EPTs compared with the control group (no intervention). Kim et al. [16] also found EPTs were significantly superior in promoting neurogenesis, via increases in number of DCX+, BrdU+, NeuN+ cells, compared with YPTs. Several of these studies [14, 16, 36] found significant increases in neurogenesis coincided with significant increases in BDNF following EPTs.

Kim et al. [16] reported significant improvements in mitochondrial function after EPTs, shown by an increase in mitochondrial Ca^2+^ retention capacity and a decrease in hydrogen peroxide (H_2_O_2_) emission rate in the hippocampus. Kim et al. [16] also reported EPTs significantly supressed apoptotic proteins (Bax, cytochrome c, apaf-1 and cleaved caspase-3 (CC3) and 9) and increased expression of Bcl-2. Concluding, EPTs significantly suppressed apoptosis compared with YPTs and controls (no intervention) [16]. Similarly, Ghaffari-Nasab et al. [18] showed that YPTs can significantly decrease apoptotic cells (CC3 and Terminal deoxynucleotidyl transferase dUTP nick end labelling (TUNEL)- positive cells) in aged animal models compared with controls (no intervention). Conversely, Kim et al. [16] found no significant difference in suppression of apoptosis between YPT and control (no intervention) groups.

#### Quality of life

Sha et al. [23, 24] found a significant improvement in quality of life after YPTs in people with AD. Quality of life was assessed using the Alzheimer Disease Cooperative Study Activities of Daily Living Inventory in mild cognitive impairment (ADCS-MCI-ADL) and Functional Activities Questionnaire (FAQ) [24]. Parker et al. [23] assessed quality of life using the Parkinson’s disease questionnaire (PDQ39) and the Unified Parkinson's Disease Rating Scale (UPDRS III). With no significant changes reported in PDQ39 total score or blinded UPDRS III [23]. However, significant improvements were reported in the stigma sub score of the PDQ39 and the unblinded UPDRS III [23].

*Review question 2:*

*What is known about the safety and tolerability of young or exercised plasma transfusions in people and animal models with neurodegenerative conditions, and what physiological parameters are measured to monitor the safety and tolerability of this therapy^+^*

*How were the safety and tolerability of plasma transfusions monitored^+^*

Of the three studies with human participants [6, 23, 24], two studies observed [23, 24], recorded and tracked adverse events, the other study as above [6] has stated they will also track adverse events. Two of these studies employed [23] or will employ [6] a safety committee, consisting of a neurologist and a transfusion medicine specialist, to review any adverse events and determine whether it is appropriate to continue treatment. Two studies [23, 24] measured vital signs, including temperature and blood pressure, pre- and post-transfusion. Tari et al. [6] will perform echocardiography to monitor heart health and to screen for patients with reduced cardiac function, participants will be excluded if found to have impaired cardiac function due to the risk of heart failure triggered by fluid overload.

#### Safety and tolerability of plasma transfusions

Five studies investigated or reported on the safety and/or tolerability of YPTs [17, 18, 23-25] and/or EPTs [17] in people and animal models with neurogenerative conditions (PD [23], AD [6, 17, 24, 25], or aged [18]). All five studies reported no serious adverse events related to the YPTs or EPTs in either human participants [23, 24] or animal models [17, 18, 25]. Using echocardiography, Norevik and collaborators [17] showed no volume overload or other harmful effects on the heart after YPTs/EPTs in AD rat models. The other two animal model studies [18, 25] used observation and weight change as a measure of tolerability and an indication of general health; both studies reported no significant differences in weight change between AD mice [25]/aged rats [18] administered YPTs or controls (no intervention [18, 25]).

In the two completed studies with human participants [23, 24], both studies reported only minor to moderate adverse reactions to YPTs (Table 3). Parker and collaborators [23] reported mild skin reactions (including bruising, itching or hives, etc) as the most experienced adverse reaction by participants. Parker et al. [23] reported one incidence of hypotension and two incidences of a cough, which were classified as possibly related to the YPTs. Parker et al. [23] found YPTs to have no adverse effects on complete blood count, chemistries, and liver and kidney function tests. Sha and collaborators [24] reported hypertension, sinus bradycardia, headache, sinus tachycardia, and dizziness as the most experienced minor to moderate adverse events. However, it was noted that there was no significant difference in occurrence of any of these adverse events between the plasma treatment group and the control (no intervention) group [24].

#### Tolerability

Both studies in humans [23, 24] used adherence to the total number of prescribed treatment sessions to assess the tolerability of YPTs. Sha et al. [24] reported a mean visit adherence of 86% across both plasma and placebo groups, while adherence after removing patients that discontinued early was 96%. Similarly, Parker and collaborators [23] mean adherence rate was 96%, and 100% after removing the sole patient who voluntarily withdrew early. Both studies concluded that YPTs were safe, feasible and well tolerated in people with PD [23] or AD [24].

*Question 3:*

*What are the protocols implementing young or exercised plasma transfusions in people or animal models with neurodegenerative conditions (intervention duration, dosage, etc)^+^*

*Protocols*

#### Alzheimer’s disease

Two animal model studies had an intervention period of 28 days or less [20, 21], Five animal model studies had an intervention period longer than 28 days [15-17, 25, 33]. In human AD trials, intervention periods were 28 days [24] and will be one year (3x 4 week periods)[6]). In animal AD studies dosages were 100 μl [16, 33]), 150 μl [20, 21, 25], or 2ml/kg body weight [15, 17]. The number of transfusions were eight [20, 21]), 10 [16, 33], 14 [15, 17], or 16 [25]. In human AD trials, Sha et al. [24] administered 250ml across four transfusions and Tari et al. [6] will administer 200ml across 12 transfusions.

**Table 3:** Related adverse events after young plasma transfusions in human participants^1^

Adverse events	
	Number of incidents (No. of patients)
**Likely related**	
**Mild skin reactions**	Bruising 1 event (1/16 patients) [23] Skin and subcutaneous tissue disorder 13 events (NS/16 patients) [23]
**Hypertension**	5 events (3/17 patients) [24]
**Dizziness**	2 events (2/17 patients) [24]
**Sinus bradycardia**	3 events (3/17 patients) [24]
**Headache**	3 events (3/17 patients) [24]
**Sinus tachycardia**	3 events (3/17 patients) [24]
**Possibly related**
**Hypotension**	1 event (1/16 patients) [23]
**Cough**	2 events (NS/16 patients) [23]

(1) Only YPTs as no EPTs studies with human participants completed. NS= Not specified

#### Aged individuals

All seven animal model studies investigating aged individuals had an intervention period of less than 28 days [14, 18, 19, 32, 34-36, 38]. In aged animal models, dosages were 100 μl [14, 34-36, 38], 150 μl [19], 175 μl [32], or 1 ml [18]). The number of transfusions were 6-8 [32], 8 [14, 34-36, 38], 7-9 [19], or 12 [18].

Of the remaining studies, one study [37] investigated EPTs in animals with LPS induced neuroinflammation which had an intervention period of 6 hrs, and a dosage of 200 μl across three transfusions. The other study [23] investigated YPTs in people with PD which had an intervention period of 28 days, and a dosage of 200ml across four transfusions.

*Question 4:*

*What is the cost effectiveness of young or exercised plasma transfusion in people with neurodegenerative conditions^+^*

None of the included studies investigated the cost effectiveness of YPTs or EPTs.

*Question 5:*

*What are the barriers and facilitators to effective implementation of plasma transfusions (young or exercised) in people or animal models with neurodegenerative conditions^+^*

None of the included studies investigated the barriers or facilitators of YPTs or EPTs.

## DISCUSSION

To our knowledge, this is the first study to review the impacts of plasma (young or exercised) transfusions in neurodegenerative conditions. Overall, the results indicate that YPTs and EPTs can both exert positive impacts on cognition (memory/learning), neuro-inflammation, neurogenesis, synaptic function and synaptic proteins, and apoptosis in neurodegenerative conditions. Additionally, early evidence suggests that YPTs and/or EPTs may improve QOL, psychiatric symptoms, mitochondrial function and microglial morphology, and play a role in reduction of Aβ and/or tau. Furthermore, some preliminary evidence suggests that EPTs may confer some/more of the systemic benefits of exercise in animal models with neurodegenerative conditions compared to YPTs. However, due to the limited and heterogenous results across studies, more research is needed to clarify exactly, and to what degree, these types of plasma transfusions can positively impact neurodegenerative conditions.

### Cognition

Significant improvements in cognition or cognitive impairments after plasma transfusions (YPTs [21, 25, 32-36]; EPTs [14, 16, 37]) were commonly reported. Cognition (memory and learning) is negatively impacted in many neurodegenerative conditions and is considered a major risk factor for morbidity and mortality [39]. Furthermore, studies show that exercise can elicit positive benefits on cognition and cognitive impairment in neurodegenerative conditions [40-42]. These results suggest that YPTs and EPTs may confer some of the benefits of exercise in this population. However, some studies that investigated these factors found no significant differences (YPTs [15, 16, 20, 23, 24], EPTs [15, 17]) between groups (No intervention [15-17, 20, 24], YPTs [15, 17]) or compared to baseline measures [23]). The variance in results has been postulated to be a result of insufficient length of intervention and/or dosage [20]. Additionally, the choice and sensitivity of the chosen cognitive tests are important considerations when analysing the variance of the results [43]. As some cognitive tests may fail to detect improvements even when an improvement in cognition has occurred [43]. In addition to the positive impacts seen in cognition, preliminary evidence indicates YPTs can also elicit significant improvements in psychiatric symptoms/mood [18, 35] and QOL measures [24]. Given the negative effects neurodegenerative conditions have on cognition and mental health [18, 39], the improvements seen after plasma transfusions could have a significant impact on overall QOL in individuals with neurodegenerative conditions [24].

### Neuroinflammation

Many of the studies that investigated neuroinflammation found positive impacts on key inflammatory proteins after treatment with either YPTs [23, 25, 38] or EPTs [15, 17, 37]. These beneficial changes were observed across multiple neurodegenerative conditions including adults with PD [23], AD animal models [15, 17, 25], LPS animal models [37], and aged animal models [38]. This is a potentially important finding as there is an increasing body of evidence showing that neuroinflammation is a key player in neurodegenerative disease initiation and pathophysiology [44]. Many of these studies [15, 23, 38] suggested that the favourable effects on inflammation observed were due to a reduction in TNF/TNF-α, which has been postulated to have an anti-inflammatory effect in neurodegenerative conditions [23]. The downregulation of inflammation suggests that the positive impacts of YPTs and EPTs are at least partly mediated by blood borne factors [6, 32]. This could counteract the adverse effects of neuroinflammation (neuronal damage) seen in neurodegenerative conditions [6, 32]. It is theorised that the accumulation of Aβ and tau seen in AD pathology is exacerbated by chronic inflammation (neuro-inflammation) and thus could be an appropriate target for therapeutic interventions [45]. In the present study several of the included studies investigated the impacts on Aβ and tau in AD [15-17, 20, 21, 25, 33]. While some of these studies found improvements [15, 20, 25, 33] after plasma transfusion, multiple studies [15-17, 21] did not observe significant impacts on these proteins. Thus, it is unclear exactly how plasma transfusions can impact these proteins and the role they play in neuroinflammation. It is theorised that an intervention period of ≤4 weeks may not be sufficient to induce significant changes in these proteins and thus explain the variation in results [17, 20]. Alternatively, Kim et al. [16] suggests improvements seen after plasma transfusions may work via a tau independent mechanism, given other improvements (cognition, neurogenesis, etc) are still seen despite minimal changes in tau [16].

### Synaptic function and protein expression

Another commonly demonstrated impact of YPTs and EPTs was an improvement in synapses and/or key synaptic proteins [14, 16, 21, 33, 34, 36]. All studies [14, 16] that investigated the impact of EPTs on BDNF found significant improvements relative to control groups (no intervention [16] or sedentary plasma [14]); increases in BDNF has been shown to correlate with improvements in neuronal survival, growth, and plasticity [46]. Exercise has also been shown to increase blood BDNF levels [46]. This provides further evidence that plasma transfusions may confer some of the positive benefits of exercise into individuals with neurodegenerative conditions. Additionally, one study [16] found EPTs to increase mitochondrial function, which can be stimulated by BDNF. Impaired regulation of mitochondrial Ca^2+^ homeostasis, via elevated levels of reactive oxygen species (including H_2_O_2_), promotes apoptosis by increasing sensitivity of mitochondrial permeability transition pore opening [16]. Therefore, improvements in mitochondrial function may directly counteract the neuronal damage caused by mitochondrial dysfunction [16]. Potentially indicating another positive impact of exercise that may be conferred via EPT. It has been shown that the cognitive impairments experienced in many neurodegenerative conditions are, in part, a result of synaptic loss and dysfunction [33]. Thus, therapies that may result in restoration and/or preservation of synapses and synaptic function, like YPTs and EPTs, warrant further investigation. However, not all studies reported significant beneficial impacts on all these synaptic proteins. Kim et al. [16] and Zhao et al. [25] found no significant differences between YPTs and controls (no intervention [16, 25]) on levels of synaptophysin [16, 25], PSD95 [16] and/or CREB [25]. Interestingly, all EPT studies found significant beneficial differences in the synaptic proteins they investigated (Synaptophysin [16], BDNF [14, 16], PSD95 [16]) relative to control groups (no intervention [16], sedentary plasma [14]), suggesting EPTs may have additional properties that result in greater impacts on key synaptic proteins in neurodegenerative conditions.

### Neurogenesis and suppression of apoptosis

Decreased cognitive function is associated with decreased neurogenesis [16]. Conflicting results were found when investigating YPTs and neurogenesis. Only two [19, 36] out of three [25] YPT studies showed significant positive impacts on neurogenesis (via changes in DCX+ or NeuN [25, 36] or downregulation of DEGs in aNSCs [19]) when compared with controls (no intervention [19, 25, 36]), whereas all EPT studies that investigated neurogenesis showed significant positive impacts (via increases in DCX+ [14, 16, 37], BrdU+ [14, 16, 17], and/or NeuN+ [14, 16, 17]) compared to controls (no intervention [16, 17], or sedentary plasma [14, 37]). Furthermore, Kim et al. [16] found EPTs significantly increased neurogenesis, via increases in DCX+, BrdU+, and NeuN+ cells, when compared with YPTs. This is an important finding as an increase in neurogenesis is linked to improved cognition, which may be a critical therapeutic target given the role cognitive impairment plays in morbidity and mortality risk [39].

Two studies showed that YPTs [18] and EPTs [16] can positively impact apoptosis, via a reduction in apoptotic proteins. Moreover, Kim et al. [16] reported EPTs were significantly superior to YPTs in suppressing apoptosis. Thus it appears that not only can YPTs/EPTs influence neurogenesis it may also rescue or prevent cell death [16, 18]. These therapies can promote brain health by supporting cell proliferation, survival and reducing neuronal death, all of which are incredibly important processes in neurodegenerative conditions [18, 46].

### Safety and feasibility

An important consideration in any potential treatment is safety [23, 24]. All included studies that investigated safety concluded that YPTs [18, 23-25] and EPTs [17] are safe, [23, 24] reporting no serious adverse events [17, 18, 23-25]. However, some mild to moderate adverse events occurred in both human trials [23, 24] (including mild skin reactions, hyper/hypotension, cough, dizziness, sinus tachy/bradycardia, and headaches). Therefore, the preliminary data suggests that with appropriate monitoring, transfusion risks can be managed and mitigated [23, 24].

Tolerability and feasibility are important considerations for any potential therapy [23, 24]. Both human trials reported high adherence rates >96% [24] and 100% [23] respectively, indicating good tolerability. However, none of the included studies investigated the barriers, facilitators, and/or cost effectiveness of YPTs and EPTs. Thus, the overall feasibility is yet to be completely determined. However, a recent study [47] showed that home-based blood (including plasma) transfusions are both safe and feasible. This is an important finding as difficulties attending health care facilities is a commonly cited barrier to receiving healthcare for people with neurodegenerative conditions [11-13]. Therefore, a home-based transfusion service could minimise some of the barriers to accessing healthcare in this population. However, more research into the barriers, facilitators, and cost effectiveness is needed before this can be conclusively determined.

Another potentially key factor in the feasibility of YPTs and EPTs as a treatment option is the availability of clinical plasma [48, 49]; many countries have low donation rates which leads to limited blood and plasma supplies [48, 49]. Further to this plasma-based interventions have regulatory and ethical considerations given the known risks and the uncertainty of the benefits [50, 51]. Thus, even if the positive impacts of YPTs/EPTs are confirmed in the future, the feasibility of implementing these therapies widely must be considered given the estimated hundreds of millions of people with neurodegenerative conditions [52]. However, as there is a severe lack of disease modifying therapies YPTs/EPTs still warrant further investigation in neurodegenerative conditions [6]. Alternatively understanding the molecular mediators of the beneficial impacts of YPTs/EPTs may overcome the issues surrounding the clinical application of plasma transfusions. This could lead to development of therapies/treatments that harness the beneficial components of YPTs/EPTs without the need to utilise already limited plasma supplies [48, 49]. The beneficial impacts of plasma transfusions appear to be multifaceted and not a result of individual factors [32, 33]. Several studies have investigated the underlying molecular mediators to explain the mechanism behind the potential beneficial impacts of YPTs and EPTs [14, 15, 25, 32, 33, 37]. These include but not limited to growth differential factor 11 (GDF11) [53], vascular endothelial growth factor (VEGF) [15], Gpld1 [14], REST/ FOXO1 [33], tissue inhibitor of metalloproteinases 2 (TIMP2) [32], and oxytocin [54, 55]. GDF11, a circulating factor in young blood, has been connected to improvements in neurogenesis [53]. Increases in VEGF levels after exercise and EPTs have been linked to improvements in neurogenesis and angiogenesis, thus providing another possible mechanism for the beneficial impacts of plasma transfusions [15]. Gpld1, a GPI- degrading enzyme upregulated in plasma after exercise, was found to alter downstream signalling cascades of GPI-anchored substrate cleavage resulting in amelioration of age-related cognitive impairments [14]. Xia et al. [33] reported the beneficial impacts they found after YPTs were in part mediated by the activation of REST/FOXO1 signalling resulting in a neuroprotective mechanism that attenuated deficits in cognition. Castellano et al. [32] found that administration of TIMP2, an upregulated blood borne factor in young plasma, improves cognition and increase synaptic plasticity in aged mice. Oxytocin has been shown to potentially have an antioxidant and trophic, anti-inflammatory, and anti-apoptotic effects in animal models which can act as a neuroprotective mechanism and reduce cell death [54, 55]. However, while administering some of these factors in isolation appears to induce positive impacts, they are predominantly at a reduced capacity compared to whole plasma transfusions [32, 33]. Still the molecular mediators of YPTs/EPTs impacts warrant further investigation, as the development of therapies/treatments that harness the beneficial components of YPTs/EPTs could overcome the issues with transfusion of young and exercised plasma.

### Strength and limitations

The present study is the first to review the impacts of plasma transfusions (young or exercised) in people and animal models with neurodegenerative conditions. This summation of the impacts, risks, and intervention characteristics of YPTs and EPTs will aid the development of potentially safer and more effective study designs. In turn, this will further progress the research into YPTs and EPTs and its use in neurodegenerative conditions. Another strength of the present study was that it was conducted in accordance with a priori protocol [27], the JBI methodology for scoping reviews [28], and the PRISMA-Scr guidelines (Appendix I)[29]. A comprehensive database search was completed, and the key findings of each included study was presented in tabular form (Table 1).

There are several limitations to consider. The included clinical studies did not report the method of allocation, blinding of researchers and participants and point and variability measures. Many of the included animal model studies did not report the method of random sequence generation, allocation concealment, random housing, blinding, and complete outcome data. Collectively this could lead to bias affecting the outcomes of this scoping review. Further to this, only studies in English were considered for this review. Several studies (n= 21) had an abstract translated to English which made the full text review stage, though were later excluded due to the full text not being in English. Thus, it is possible that relevant studies have been excluded.

### Implications for research

The current review summaries the available research on two novel therapy types (YPTs/EPTs) in people and animal models with neurodegenerative conditions. The preliminary evidence suggests that YPTs and EPTs can exert positive impacts on neurodegenerative conditions. However, this review demonstrates that the currently available research is limited. Research is needed in multiple areas with the gaps in the literature and future recommendations summarised in Table 4.

**Table 4:** Identified gaps in the literature and future recommendations

Identified gaps in included studies	Future recommendations
**Currently it is unclear why there are conflicting results between studies. A proposed explanation of the variance could be attributed to the heterogeneity in participant characteristics, transfusion dosage, length of intervention and type of plasma used [16, 20]. It has been suggested that some of potential beneficial impacts of plasma transfusions are not realised in studies with shorter intervention durations (< 8 weeks) [17, 20, 21, 24]. Another explanation of the variance in results is the timing of therapies in relation to disease stage (early compared to late-stage disease) [15]. Other potential causes of the heterogeneity in results include pharmacokinetics and pharmacodynamics, small sample sizes, lack of blinding of participants/researchers, and study designs that were not powered to determine the impacts of plasma transfusions.**	Conduct studies with: 1.Larger sample sizes and longer intervention trials (≥ 8 weeks) to ascertain if the variance in results are due to the length of intervention or dosage of plasma. 2.Study designs that minimise the impact of heterogeneity of participant characteristics at baseline (e.g. double blinded randomised crossover trials). 3.Participants at different stages of disease progression (e.g. early compared to late-stage disease) to explore the impact of timing of plasma transfusion therapy. 4.Other potential sources of limitations considered including medications, blinding of participants/researchers, and study designs geared to determine the impacts of YPTs/EPTs
**Currently there is a limited number of human trials (n= 2) investigating YPTs/EPTs in neurodegenerative conditions. Furthermore, the primary aim in these studies was to assess safety and tolerability of YPTs, with assessment of impacts as secondary exploratory outcomes, with little methodological consistency and poor to fair methodological quality. Thus, the impacts of YPTs in people with neurodegenerative conditions is still unclear.**	1.Robust double blind randomised crossover trials to assess the impacts, safety, and feasibility of YPTs and/or EPTs in people with neurodegenerative conditions that are powered to determine clinical and statistical significance 2.Studies that investigate the impacts of YPTs compared to EPTs to ascertain the efficacy of each plasma type
**Currently no research has been published on the impacts of EPTs in people with neurodegenerative conditions. Only three animal model studies [15-17] compared the impacts of YPTs compared to EPTs. Therefore, it is not yet clear whether the impacts of EPTs are comparable or superior to YPTs and/or applicable to people with neurodegenerative conditions.**	
**Whether the potential benefits of YPTs/EPTs are a result of the removal of negative factors or addition of beneficial factors or a combination of both[20].**	Future studies investigating YPTs and EPTs should seek to understand the underlying mechanisms and molecular mediators underpinning potential impacts to aid in development of therapies/treatments that harness the beneficial components of YPTs/EPTs without the need to utilise already limited plasma supplies
**The existing body of research primarily focuses on AD (n= 9) or aged participants (n= 8) (Table 1), which may limit the applicability of the results to other neurodegenerative conditions.**	Investigate the impacts, safety, and feasibility of YPTs and EPTs in other neurodegenerative conditions

## Conclusion

Overall, both YPTs and EPTs have been shown to have beneficial impacts in people and animal models with neurodegenerative conditions. Beneficial impacts on cognition, neuroinflammation, neurogenesis, apoptosis, and synaptic function/synaptic proteins were commonly reported. Given the lack of disease modifying therapies available for neurodegenerative conditions both plasma types warrant further investigation. However, due to the limited and heterogenous results across studies more research is needed to ascertain the exact impacts, safety, mechanisms, and feasibility of YPTs and EPTs. Furthermore, given the regulatory, ethical and scalability concerns of using plasma transfusions as a therapy. Future research into plasma transfusions should be focused on understanding the underlying mechanisms that are responsible for the beneficial impacts. Ultimately this could lead to the development of therapies that replicate the benefits of YPTs and EPTs without many of the current issues surrounding plasma transfusions and supply.

## Supplementary Materials

The Supplementary data can be found online at: www.aginganddisease.org/EN/10.14336/AD.2025.0849.

## Data Availability

All data that is relevant to the study are included within the article.

## References

[1] Zaib S, Javed H, Khan I, Jaber F, Sohail A, Zaib Z, et al. (2023). Neurodegenerative diseases: their onset, epidemiology, causes and treatment. ChemistrySelect, 8:e202300225.

[2] Edgren G, Hjalgrim H, Rostgaard K, Lambert P, Wikman A, Norda R, et al. (2016). Transmission of neurodegenerative disorders through blood transfusion: a cohort study. Annals of internal medicine, 165:316-324. 10.7326/M15-2421 27368068

[3] Lamptey RNL, Chaulagain B, Trivedi R, Gothwal A, Layek B, Singh J (2022). A Review of the Common Neurodegenerative Disorders: Current Therapeutic Approaches and the Potential Role of Nanotherapeutics. Int J Mol Sci, 23. 10.3390/ijms23031851 PMC8837071 35163773

[4] Feigin VL, Vos T, Nichols E, Owolabi MO, Carroll WM, Dichgans M, et al. (2020). The global burden of neurological disorders: translating evidence into policy. Lancet Neurol, 19:255-265. 10.1016/S1474-4422(19)30411-9 PMC9945815 31813850

[5] Steinmetz JD, Seeher KM, Schiess N, Nichols E, Cao B, Servili C, et al. (2024). Global, regional, and national burden of disorders affecting the nervous system, 1990-2021: a systematic analysis for the Global Burden of Disease Study 2021. The Lancet Neurology, 23:344-381. 10.1016/S1474-4422(24)00038-3 PMC10949203 38493795

[6] Tari AR, Berg HH, Videm V, Bråthen G, White LR, Røsbjørgen RN, et al. (2022). Safety and efficacy of plasma transfusion from exercise-trained donors in patients with early Alzheimer’s disease: protocol for the ExPlas study. BMJ open, 12:e056964. 10.1136/bmjopen-2021-056964 PMC9453994 36538409

[7] Adams ML, Grandpre J, Katz DL, Shenson D (2019). The impact of key modifiable risk factors on leading chronic conditions. Preventive Medicine, 120:113-118. 10.1016/j.ypmed.2019.01.006 30658065

[8] Elagizi A, Kachur S, Carbone S, Lavie CJ, Blair SN (2020). A Review of Obesity, Physical Activity, and Cardiovascular Disease. Current Obesity Reports, 9:571-581. 10.1007/s13679-020-00403-z 32870465

[9] Alizaei Yousefabadi H, Niyazi A, Alaee S, Fathi M, Mohammad Rahimi GR (2021). Anti-Inflammatory Effects of Exercise on Metabolic Syndrome Patients: A Systematic Review and Meta-Analysis. Biological Research For Nursing, 23:280-292. 10.1177/1099800420958068 32938197

[10] de Sousa Fernandes MS, Ordônio TF, Santos GCJ, Santos LER, Calazans CT, Gomes DA, et al. (2020). Effects of Physical Exercise on Neuroplasticity and Brain Function: A Systematic Review in Human and Animal Studies. Neural Plasticity, 2020:8856621. 10.1155/2020/8856621 PMC7752270 33414823

[11] Ellis T, Boudreau JK, DeAngelis TR, Brown LE, Cavanaugh JT, Earhart GM, et al. (2013). Barriers to Exercise in People With Parkinson Disease. Physical Therapy, 93:628-636. 10.2522/ptj.20120279 PMC3641403 23288910

[12] Mulligan HF, Hale LA, Whitehead L, Baxter GD (2012). Barriers to Physical Activity for People With Long-Term Neurological Conditions: A Review Study. Adapted Physical Activity Quarterly, 29:243-265. 10.1123/apaq.29.3.243 22811565

[13] Schootemeijer S, Van Der Kolk NM, Ellis T, Mirelman A, Nieuwboer A, Nieuwhof F, et al. (2020). Barriers and motivators to engage in exercise for persons with Parkinson’s disease. Journal of Parkinson's disease, 10:1293-1299. 10.3233/JPD-202247 PMC7739964 32925106

[14] Horowitz AM, Fan X, Bieri G, Smith LK, Sanchez-Diaz CI, Schroer AB, et al. (2020). Blood factors transfer beneficial effects of exercise on neurogenesis and cognition to the aged brain. Science, 369:167-173. 10.1126/science.aaw2622 PMC7879650 32646997

[15] Huuha AM, Norevik CS, Coombes JS, Røsbjørgen RN, Miguel-Dos-Santos R, Moreira JBN, et al. (2024). Effects of Intravenously Administered Plasma from Exercise-Trained Donors on Microglia and Cytokines in a Transgenic Rat Model of Alzheimer's Disease. Brain Plast, 9:21-41. 10.3233/BPL-230154 PMC11234670 38993579

[16] Kim TW, Park SS, Park JY, Park HS (2020). Infusion of Plasma from Exercised Mice Ameliorates Cognitive Dysfunction by Increasing Hippocampal Neuroplasticity and Mitochondrial Functions in 3xTg-AD Mice. International Journal of Molecular Sciences, 21. 10.3390/ijms21093291 PMC7247545 32384696

[17] Norevik CS, Huuha AM, Røsbjørgen RN, Hildegard Bergersen L, Jacobsen K, Miguel-dos-Santos R, et al. (2024). Exercised blood plasma promotes hippocampal neurogenesis in the Alzheimer's disease rat brain. Journal of Sport and Health Science, 13:245-255. 10.1016/j.jshs.2023.07.003 PMC10980897 37500010

[18] Ghaffari-Nasab A, Badalzadeh R, Mohaddes G, Alipour MR (2021). Young plasma administration mitigates depression-like behaviours in chronic mild stress-exposed aged rats by attenuating apoptosis in prefrontal cortex. Exp Physiol, 106:1621-1630. 10.1113/EP089415 34018261

[19] Hahn O, Foltz AG, Atkins M, Kedir B, Moran-Losada P, Guldner IH, et al. (2023). Atlas of the aging mouse brain reveals white matter as vulnerable foci. Cell, 186:4117-4133.e4122. 10.1016/j.cell.2023.07.027 PMC10528304 37591239

[20] Hernandez CM, Barkey RE, Craven KM, Pedemonte KA, Alisantosa B, Sanchez JO, et al. (2023). Transfusion with Blood Plasma from Young Mice Affects rTg4510 Transgenic Tau Mice Modeling of Alzheimer's Disease. Brain Sci, 13. 10.3390/brainsci13060841 PMC10296323 37371321

[21] Middeldorp J, Lehallier B, Villeda SA, Miedema SS, Evans E, Czirr E, et al. (2016). Preclinical Assessment of Young Blood Plasma for Alzheimer Disease. JAMA Neurol, 73:1325-1333. 10.1001/jamaneurol.2016.3185 PMC5172595 27598869

[22] Li N, Zhang X, Gu Z, Su C, Lian H (2021). Young plasma attenuates cognitive impairment and the cortical hemorrhage area in cerebral amyloid angiopathy model mice. Ann Transl Med, 9:147. 10.21037/atm-20-8008 PMC7867917 33569449

[23] Parker JE, Martinez A, Deutsch GK, Prabhakar V, Lising M, Kapphahn KI, et al. (2020). Safety of Plasma Infusions in Parkinson's Disease. Movement Disorders. 10.1002/mds.28198 PMC7361360 32633860

[24] Sha SJ, Deutsch GK, Tian L, Richardson K, Coburn M, Gaudioso JL, et al. (2019). Safety, Tolerability, and Feasibility of Young Plasma Infusion in the Plasma for Alzheimer Symptom Amelioration Study: A Randomized Clinical Trial. JAMA Neurology, 76:35-40. 10.1001/jamaneurol.2018.3288 PMC6439869 30383097

[25] Zhao Y, Qian R, Zhang J, Liu F, Iqbal K, Dai CL, et al. (2020). Young blood plasma reduces Alzheimer's disease-like brain pathologies and ameliorates cognitive impairment in 3×Tg-AD mice. Alzheimers Res Ther, 12:70. 10.1186/s13195-020-00639-w PMC7278124 32513253

[26] Kohli R, Geneen LJ, Brunskill SJ, Doree C, Estcourt L, Chee SEJ, et al. (2022). Assessing efficacy and safety of replacement fluids in therapeutic plasma exchange: A systematic scoping review of outcome measures used. J Clin Apher, 37:438-448. 10.1002/jca.21996 PMC9795884 35726507

[27] Kirton MJ, Cox ER, Dalleck L, Lange B, Drummond C, Chakraborty R, et al. (2025). The Impact of Young and/or Exercised Blood Plasma Transfusions in Individuals With Neurodegenerative Conditions: Protocol for a Scoping Review. JMIR Research Protocols, 14:e65935. 10.2196/65935 PMC12405790 40829149

[28] Munn Z, Aromataris E, Tufanaru C, Stern C, Porritt K, Farrow J, et al. (2019). The development of software to support multiple systematic review types: the Joanna Briggs Institute System for the Unified Management, Assessment and Review of Information (JBI SUMARI). Int J Evid Based Healthc, 17:36-43. 10.1097/XEB.0000000000000152 30239357

[29] Page MJ, McKenzie JE, Bossuyt PM, Boutron I, Hoffmann TC, Mulrow CD, et al. (2021). The PRISMA 2020 statement: an updated guideline for reporting systematic reviews. BMJ, 372:n71. 10.1136/bmj.n71 PMC8005924 33782057

[30] Hooijmans CR, Rovers MM, De Vries RB, Leenaars M, Ritskes-Hoitinga M, Langendam MW (2014). SYRCLE’s risk of bias tool for animal studies. BMC medical research methodology, 14:43. 10.1186/1471-2288-14-43 PMC4230647 24667063

[31] Maher CG, Sherrington C, Herbert RD, Moseley AM, Elkins M (2003). Reliability of the PEDro scale for rating quality of randomized controlled trials. Physical therapy, 83:713-721. 12882612

[32] Castellano JM, Mosher KI, Abbey RJ, McBride AA, James ML, Berdnik D, et al. (2017). Human umbilical cord plasma proteins revitalize hippocampal function in aged mice. Nature, 544:488-492. 10.1038/nature22067 PMC5586222 28424512

[33] Xia E, Xu F, Hu C, Kumal JPP, Tang X, Mao D, et al. (2019). Young Blood Rescues the Cognition of Alzheimer's Model Mice by Restoring the Hippocampal Cholinergic Circuit. Neuroscience, 417:57-69. 10.1016/j.neuroscience.2019.08.010 31404586

[34] Villeda SA, Plambeck KE, Middeldorp J, Castellano JM, Mosher KI, Luo J, et al. (2014). Young blood reverses age-related impairments in cognitive function and synaptic plasticity in mice. Nature Medicine, 20:659-663. 10.1038/nm.3569 PMC4224436 24793238

[35] Khrimian L, Obri A, Ramos-Brossier M, Rousseaud A, Moriceau S, Nicot AS, et al. (2017). Gpr158 mediates osteocalcin's regulation of cognition. J Exp Med, 214:2859-2873. 10.1084/jem.20171320 PMC5626410 28851741

[36] Smith L.2020. Systemic Regulation of Synaptic, Regenerative, and Cognitive Dysfunction During Aging.

[37] De Miguel Z, Khoury N, Betley MJ, Lehallier B, Willoughby D, Olsson N, et al. (2021). Exercise plasma boosts memory and dampens brain inflammation via clusterin. Nature, 600:494-499. 10.1038/s41586-021-04183-x PMC9721468 34880498

[38] Schroer AB, Ventura PB, Sucharov J, Misra R, Chui MKK, Bieri G, et al. (2023). Platelet factors attenuate inflammation and rescue cognition in ageing. Nature, 620:1071-1079. 10.1038/s41586-023-06436-3 PMC10468395 37587343

[39] Trojsi F, Christidi F, Migliaccio R, Santamaría-García H, Santangelo G (2018). Behavioural and Cognitive Changes in Neurodegenerative Diseases and Brain Injury. Behav Neurol, 2018:4935915. 10.1155/2018/4935915 PMC6083604 30147810

[40] Murray DK, Sacheli MA, Eng JJ, Stoessl AJ (2014). The effects of exercise on cognition in Parkinson’s disease: a systematic review. Translational Neurodegeneration, 3:5. 10.1186/2047-9158-3-5 PMC3936925 24559472

[41] Fernandes RM, Correa MG, dos Santos MAR, Almeida APCPSC, Fagundes NCF, Maia LC, et al. (2018). The Effects of Moderate Physical Exercise on Adult Cognition: A Systematic Review. Frontiers in Physiology, 9 -2018. 10.3389/fphys.2018.00667 PMC6002532 29937732

[42] Farì G, Lunetti P, Pignatelli G, Raele MV, Cera A, Mintrone G, et al. (2021). The Effect of Physical Exercise on Cognitive Impairment in Neurodegenerative Disease: From Pathophysiology to Clinical and Rehabilitative Aspects. International Journal of Molecular Sciences, 22:11632. 10.3390/ijms222111632 PMC8583932 34769062

[43] Ghafarimoghadam M, Mashayekh R, Gholami M, Fereydani P, Shelley-Tremblay J, Kandezi N, et al. (2022). A review of behavioral methods for the evaluation of cognitive performance in animal models: Current techniques and links to human cognition. Physiology & Behavior, 244:113652. 10.1016/j.physbeh.2021.113652 34801559

[44] Zhang W, Xiao D, Mao Q, Xia H (2023). Role of neuroinflammation in neurodegeneration development. Signal Transduction and Targeted Therapy, 8:267. 10.1038/s41392-023-01486-5 PMC10336149 37433768

[45] Kinney JW, Bemiller SM, Murtishaw AS, Leisgang AM, Salazar AM, Lamb BT (2018). Inflammation as a central mechanism in Alzheimer's disease. Alzheimers Dement (N Y), 4:575-590. 10.1016/j.trci.2018.06.014 PMC6214864 30406177

[46] Romero Garavito A, Díaz Martínez V, Juárez Cortés E, Negrete Díaz JV, Montilla Rodríguez LM (2024). Impact of physical exercise on the regulation of brain-derived neurotrophic factor in people with neurodegenerative diseases. Front Neurol, 15:1505879. 10.3389/fneur.2024.1505879 PMC11810746 39935805

[47] Rodríguez Corte J, Candal-Pedreira C, Ruano-Ravina A, Pérez-Ríos M, Rivero-de-Aguilar A, López García M, et al. (2022). Home-based blood transfusion therapy: A systematic review. Br J Haematol, 199:496-506. 10.1111/bjh.18344 PMC9796283 35778372

[48] Strengers PFW (2023). Challenges for Plasma-Derived Medicinal Products. Transfusion Medicine and Hemotherapy, 50:116-122. 10.1159/000528959 PMC10091012 37066053

[49] Hartmann J, Klein HG (2020). Supply and demand for plasma-derived medicinal products - A critical reassessment amid the COVID-19 pandemic. Transfusion, 60:2748-2752. 10.1111/trf.16078 PMC7460929 32856742

[50] Skinner MW, Hedlund Hoppe PA, Grabowski HG, Manning R, Tachdjian R, Crone JF, et al. (2016). Risk-based decision making and ethical considerations in donor compensation for plasma-derived medicinal products. Transfusion, 56:2889-2894. 10.1111/trf.13764 27600165

[51] Codorean I-B, Cernat E, Popescu D, Sinescu RD, Perlea P (2017). Legal and ethical aspects of Platelet-Rich Plasma. Rom. J. Leg. Med, 25:405-408.

[52] Gadhave DG, Sugandhi VV, Jha SK, Nangare SN, Gupta G, Singh SK, et al. (2024). Neurodegenerative disorders: Mechanisms of degeneration and therapeutic approaches with their clinical relevance. Ageing Research Reviews, 99:102357. 10.1016/j.arr.2024.102357 38830548

[53] Katsimpardi L, Litterman NK, Schein PA, Miller CM, Loffredo FS, Wojtkiewicz GR, et al. (2014). Vascular and Neurogenic Rejuvenation of the Aging Mouse Brain by Young Systemic Factors. Science, 344:630-634. 10.1126/science.1251141 PMC4123747 24797482

[54] Erbas O, Oltulu F, Taskiran D (2013). Suppression of exaggerated neuronal oscillations by oxytocin in a rat model of Parkinson’s disease. Gen Physiol Biophys, 32:517-525. 10.4149/gpb_2013055 23970035

[55] Balmus I, Ciobica A, Stoica B, Lefter R, Cojocaru S, Reznikov A (2019). Effects of oxytocin administration on oxidative markers in the temporal lobe of aged rats. Neurophysiology, 51:18-24.

