# Supplementary material for: The Impact of Young and/or Exercised Plasma Transfusions in Neurodegenerative Conditions: A Scoping Review
Source: Aging Dis. 2026 Oct 16;17(6):2839–56. doi: 10.14336/AD.2025.0849 (PMC13653718; doi:10.14336/AD.2025.0849)
Supplement: Supplementary file 1 [file AD-17-6-2839-s.pdf]

The Impact of Young and/or Exercised Plasma Transfusions in Neurodegenerative Conditions: A Scoping Review

Michael J. Kirton, Emily R. Cox, Ulrik Wisløff, Atefe R. Tari, Lance C. Dalleck, Claire Drummond, Belinda Lange, Ranjay Chakraborty, Kym Williams, Norma Bulamu, Alline Beleigoli, Joyce S. Ramos

Supplementary data

Appendix I

Preferred Reporting Items for Systematic reviews and Meta-Analyses extension for Scoping Reviews (PRISMA-ScR) checklist

SECTION	ITEM	PRISMA-ScR CHECKLIST ITEM	REPORTED ON PAGE #
**TITLE**			
Title	1	Identify the report as a scoping review.	1
**ABSTRACT**			
Structured summary	2	Provide a structured summary that includes (as applicable): background, objectives, eligibility criteria, sources of evidence, charting methods, results, and conclusions that relate to the review questions and objectives.	1
**INTRODUCTION**			
Rationale	3	Describe the rationale for the review in the context of what is already known. Explain why the review questions/objectives lend themselves to a scoping review approach.	2-3
Objectives	4	Provide an explicit statement of the questions and objectives being addressed with reference to their key elements (e.g., population or participants, concepts, and context) or other relevant key elements used to conceptualize the review questions and/or objectives.	2-3
**METHODS**			
Protocol and registration	5	Indicate whether a review protocol exists; state if and where it can be accessed (e.g., a Web address); and if available, provide registration information, including the registration number.	3
Eligibility criteria	6	Specify characteristics of the sources of evidence used as eligibility criteria (e.g., years considered, language, and publication status), and provide a rationale.	3
Information sources*	7	Describe all information sources in the search (e.g., databases with dates of coverage and contact with authors to identify additional sources), as well as the date the most recent search was executed.	3-4
Search	8	Present the full electronic search strategy for at least 1 database, including any limits used, such that it could be repeated.	Appendix II
Selection of sources of evidence†	9	State the process for selecting sources of evidence (i.e., screening and eligibility) included in the scoping review.	4
Data charting process‡	10	Describe the methods of charting data from the included sources of evidence (e.g., calibrated forms or forms that have been tested by the team before their use, and whether data charting was done independently or in duplicate) and any processes for obtaining and confirming data from investigators.	4
Data items	11	List and define all variables for which data were sought and any assumptions and simplifications made.	4
Critical appraisal of individual sources of evidence§	12	If done, provide a rationale for conducting a critical appraisal of included sources of evidence; describe the methods used and how this information was used in any data synthesis (if appropriate).	4
Synthesis of results	13	Describe the methods of handling and summarizing the data that were charted.	4

SECTION	ITEM	PRISMA-ScR CHECKLIST ITEM	REPORTED ON PAGE #
**RESULTS**			
Selection of sources of evidence	14	Give numbers of sources of evidence screened, assessed for eligibility, and included in the review, with reasons for exclusions at each stage, ideally using a flow diagram.	Appendix IV
Characteristics of sources of evidence	15	For each source of evidence, present characteristics for which data were charted and provide the citations.	5-9
Critical appraisal within sources of evidence	16	If done, present data on critical appraisal of included sources of evidence (see item 12).	9
Results of individual sources of evidence	17	For each included source of evidence, present the relevant data that were charted that relate to the review questions and objectives.	5-13
Synthesis of results	18	Summarize and/or present the charting results as they relate to the review questions and objectives.	Table 1,2 and 3 Pages 5-12
**DISCUSSION**			
Summary of evidence	19	Summarize the main results (including an overview of concepts, themes, and types of evidence available), link to the review questions and objectives, and consider the relevance to key groups.	13-15
Limitations	20	Discuss the limitations of the scoping review process.	25-16
Conclusions	21	Provide a general interpretation of the results with respect to the review questions and objectives, as well as potential implications and/or next steps.	17
**FUNDING**			
Funding	22	Describe sources of funding for the included sources of evidence, as well as sources of funding for the scoping review. Describe the role of the funders of the scoping review.	17

JBI = Joanna Briggs Institute; PRISMA-ScR = Preferred Reporting Items for Systematic reviews and Meta-Analyses extension for Scoping Reviews.

* Where *sources of evidence* (see second footnote) are compiled from, such as bibliographic databases, social media platforms, and Web sites.

† A more inclusive/heterogeneous term used to account for the different types of evidence or data sources (e.g., quantitative and/or qualitative research, expert opinion, and policy documents) that may be eligible in a scoping review as opposed to only studies. This is not to be confused with *information sources* (see first footnote).

‡ The frameworks by Arksey and O'Malley (6) and Levac and colleagues (7) and the JBI guidance (4, 5) refer to the process of data extraction in a scoping review as data charting.

§ The process of systematically examining research evidence to assess its validity, results, and relevance before using it to inform a decision. This term is used for items 12 and 19 instead of "risk of bias" (which is more applicable to systematic reviews of interventions) to include and acknowledge the various sources of evidence that may be used in a scoping review (e.g., quantitative and/or qualitative research, expert opinion, and policy document).

Appendix II: Search Strategy

Supplementary Table 1- Development of population, concept, context search terms for preliminary search

**Population (P)**	**Concept (C)**	**Context (C)**
Neurological patients	Plasma transfusion	Impacts
Neurodegenerative	Plasma exchange	Efficacy
Neurological Models	Blood plasma transfusion	Effects
Parkinson's disease	Therapeutic plasma exchange	Tolerability
Alzheimer's disease	Blood component transfusion	Rejuvenative
Motor neuron disease	Infusion	Improve
Prion disease	Transfusion medicine	Restorative
Dementia	Plasma infusion	Symptoms
Amyotrophic lateral sclerosis	Plasmapheresis	Progression
Multiple sclerosis	Exercised plasma	Safety
Huntington's disease	Exercise trained donors	Reverse
Spinocerebellar ataxia	Young plasma	Efficacy
	Young fresh frozen plasma	Cost effectiveness
	Blood factors transfer	Use

Search strings used to search databases

PUBMED CENTRAL- 718 citations found

(((neurological[Abstract] OR neurodegenerative[Abstract] OR Parkinson's disease[Abstract] OR Alzheimer's disease[Abstract] OR Motor neuron disease[Abstract] OR Dementia[Abstract] OR Multiple sclerosis[Abstract] OR Prion disease[Abstract] OR Amyotrophic lateral sclerosis[Abstract] OR Huntington's disease[Abstract] OR Spinocerebellar ataxia[Abstract])) AND ("blood transfusion"[Abstract] OR "blood component transfusion"[Abstract] OR "plasma transfusion"[Abstract] OR plasmapheresis[Abstract] OR "plasma exchange"[Abstract] OR "plasma infusion"[Abstract] OR "transfusion medicine"[Abstract] OR "exercised plasma"[Abstract] OR "young plasma"[Abstract] OR "young fresh frozen plasma"[Abstract] OR "exercise-trained donors"[Abstract] OR "exercise trained donors"[Abstract] OR "exercise plasma"[Abstract] OR "young blood"[Abstract] OR "exercise plasma"[Abstract])) AND (restor*[Abstract] OR reverse*[Abstract] OR rejuvenat*[Abstract] OR therapeutic[Abstract] OR therap*[Abstract] OR "therapeutic effect*"[Abstract] OR safe*[Abstract] OR efficacy[Abstract] OR improve*[Abstract] OR progress*[Abstract] OR effect*[Abstract] OR tolerab*[Abstract] OR symptom*[Abstract] OR attenuate*[Abstract] OR boost*[Abstract] OR gut microbio*[Abstract] OR "gut-brain axis"[Abstract] OR "gut brain axis"[Abstract] OR mitochondria*[Abstract] OR cost[Abstract] OR "cost effective*"[Abstract] OR "cost-effective*"[Abstract]) AND (("2004/01/01"[PDat] : "2024/12/31"[PDat])) Filters: Publication date from 2004/01/01 to 2024/05/31

MEDLINE- 1661 citations found

https://access.ovid.com/custom/redirector/index.html?dest=https://go.openathens.net/redirector/flinders.edu.au?url=http://ovidsp.ovid.com/ovidweb.cgi?T=JS&NEWS=N&PAGE=main&SHAREDSEARCHID=2K6jJXoEG1p2NE3HYfD9Zi0udZXNDtBKHGFQMvC58hlK9hAofqY6OqwIZhqt6qCoX

Ovid MEDLINE(R) and Epub Ahead of Print, In-Process, In-Data-Review & Other Non-Indexed Citations, Daily and Versions <1946 to June 19, 2024>

1	(neurological or neurodegenerative).tw.	406651
2	(Parkinson's disease or Alzheimer's disease or Motor neuron disease or Dementia or Multiple sclerosis or Prion disease or Amyotrophic lateral sclerosis or Huntington's disease or Spinocerebellar ataxia).tw.	490223
3	("blood transfusion" or "blood component transfusion" or "plasma transfusion" or plasmapheresis or "plasma exchange" or "plasma infusion" or "transfusion medicine").tw.	64192
4	("exercised plasma" or "young plasma" or "young fresh frozen plasma" or "exercise-trained donors" or "exercise trained donors" or "exercise plasma" or "young blood" or "exercise plasma").tw.	892
5	(restor* or reverse* or rejuvenat* or therapeutic or therap* or "therapeutic effect*" or safe* or efficacy or improve* or progress* or effect* or tolerab* or symptom* or attenuate* or boost* or gut microbio* or "gut-brain axis" or "gut brain axis" or mitochondria* or cost or "cost effective*" or "cost-effective*").tw.	15356216
6	1 or 2	805466
7	3 or 4	65068
8	5 and 6 and 7	2090
9	limit 8 to yr="2004 -Current"	1661

Cochrane Database of Systematic Reviews- 17 citations found

17 Cochrane Reviews matching neurological or neurodegenerative or Parkinson's disease or Alzheimer's disease or Motor neuron disease or Dementia or Multiple sclerosis or Prion disease or Amyotrophic lateral sclerosis or Huntington's disease or Spinocerebellar ataxia in Title Abstract Keyword AND "blood transfusion" or "blood component transfusion" or "plasma transfusion" or plasmapheresis or "plasma exchange" or "plasma infusion" or "transfusion medicine" or "exercised plasma" or "young plasma" or "young fresh frozen plasma" or "exercise-trained donors" or "exercise trained donors" or "exercise plasma" or "young blood" or "exercise plasma" in Title Abstract Keyword AND restor* or reverse* or rejuvenat* or therapeutic or therap* or therapeutic NEXT effect* or safe* or efficacy or improve* or progress* or effect* or tolerab* or symptom* or attenuate* or boost* or gut microbio* or "gut-brain axis" or "gut brain axis" or mitochondria* or cost or cost NEXT effective* or cost-effective in Title Abstract Keyword - (Word variations have been searched)

Scopus- 355 citations found

(TITLE-ABS-KEY (neurological OR neurodegenerative OR "Parkinson’s disease" OR "Alzheimer’s disease" OR "Motor neuron disease" OR dementia OR "Multiple sclerosis" OR "Prion disease" OR "Amyotrophic lateral sclerosis" OR "Huntington* disease" OR "Spinocerebellar ataxia") AND TITLE-ABS-KEY ("blood transfusion" OR "blood component transfusion" OR "plasma transfusion" OR plasmapheresis OR "plasma exchange" OR "plasma infusion" OR "transfusion medicine" OR "exercised plasma" OR "young plasma" OR "young fresh frozen plasma" OR "exercise-trained donors" OR "exercise trained donors" OR "exercise plasma" OR "young blood" OR "exercise plasma") AND TITLE-ABS-KEY (restor* OR reverse* OR rejuvenat* OR therapeutic OR therap* OR "therapeutic effect*" OR safe* OR efficacy OR improve* OR progress* OR effect* OR tolerab* OR symptom* OR attenuate* OR boost* OR gut AND microbio* OR "gut-brain axis" OR "gut brain axis" OR mitochondria* OR cost OR "cost effective*" OR cost-effective)) AND PUBYEAR > 2003 AND PUBYEAR < 2025 AND (LIMIT-TO (LANGUAGE , "English"))

Proquest- 873 citations found

https://www.proquest.com/search/2647615?accountid=10910
summary(neurological OR neurodegenerative OR "Parkinson's disease" OR "Alzheimer's disease" OR "Motor neuron disease" OR dementia OR "Multiple sclerosis" OR "Prion disease" OR "Amyotrophic lateral sclerosis" OR "Huntington* disease" OR "Spinocerebellar ataxia") AND summary("blood transfusion" OR "blood component transfusion" OR "plasma transfusion" OR plasmapheresis OR "plasma

exchange" OR "plasma infusion" OR "transfusion medicine" OR "exercised plasma" OR "young plasma" OR "young fresh frozen plasma" OR "exercise-trained donors" OR "exercise trained donors" OR "exercise plasma" OR "young blood" OR "exercise plasma") AND summary(restor* OR reverse* OR rejuvenat* OR therapeutic OR therap* OR "therapeutic effect*" OR safe* OR efficacy OR improve* OR progress* OR effect* OR tolerab* OR symptom* OR attenuate* OR boost* OR gut AND microbio* OR "gut-brain axis" OR "gut brain axis" OR mitochondria* OR cost OR "cost effective*" OR cost-effective) AND la.exact("ENG")

SPORTdiscus-20 citations found

(neurological OR neurodegenerative OR "Parkinson's disease" OR "Alzheimer's disease" OR "Motor neuron disease" OR dementia OR "Multiple sclerosis" OR "Prion disease" OR "Amyotrophic lateral sclerosis" OR "Huntington's disease" OR "Spinocerebellar ataxia") AND ("blood transfusion" OR "blood component transfusion" OR "plasma transfusion" OR plasmapheresis OR "plasma exchange" OR "plasma infusion" OR "transfusion medicine" OR "exercised plasma" OR "young plasma" OR "young fresh frozen plasma" OR "exercise-trained donors" OR "exercise trained donors" OR "exercise plasma" OR "young blood" OR "exercise plasma") AND (restor* OR reverse* OR rejuvenat* OR therapeutic OR therap* OR "therapeutic effect*" OR safe* OR efficacy OR improve* OR progress* OR effect* OR tolerab* OR symptom* OR attenuate* OR boost* OR gut AND microbio* OR "gut-brain axis" OR "gut brain axis" OR mitochondria* OR cost OR "cost effective*" OR cost-effective)

PROSPERO-13 citations found

(neurological OR neurodegenerative OR "Parkinson's disease" OR "Alzheimer's disease" OR "Motor neuron disease" OR dementia OR "Multiple sclerosis" OR "Prion disease" OR "Amyotrophic lateral sclerosis" OR "Huntington's disease" OR "Spinocerebellar ataxia") AND ("blood transfusion" OR "blood component transfusion" OR "plasma transfusion" OR plasmapheresis OR "plasma exchange" OR "plasma infusion" OR "transfusion medicine" OR "exercised plasma" OR "young plasma" OR "young fresh frozen plasma" OR "exercise-trained donors" OR "exercise trained donors" OR "exercise plasma" OR "young blood" OR "exercise plasma") AND (restor* OR reverse* OR rejuvenat* OR therapeutic OR therap* OR "therapeutic effect*" OR safe* OR efficacy OR improve* OR progress* OR effect* OR tolerab* OR symptom* OR attenuate* OR boost* OR gut AND microbio* OR "gut-brain axis" OR "gut brain axis" OR mitochondria* OR cost OR "cost effective*" OR cost-effective)

Appendix III

Supplementary Table 2- *Modified JBI data extraction tool/Priori preliminary extraction tool*

Study #	Authors, country, and year	Aims, Purpose, Objectives	Sample size	Participants	Condition/s	Age	Type of plasma used and # of treatment sessions	Follow up duration	Key findings related to the scoping review questions

Appendix IV

Supplementary Figure 1- PRISMA flow diagram

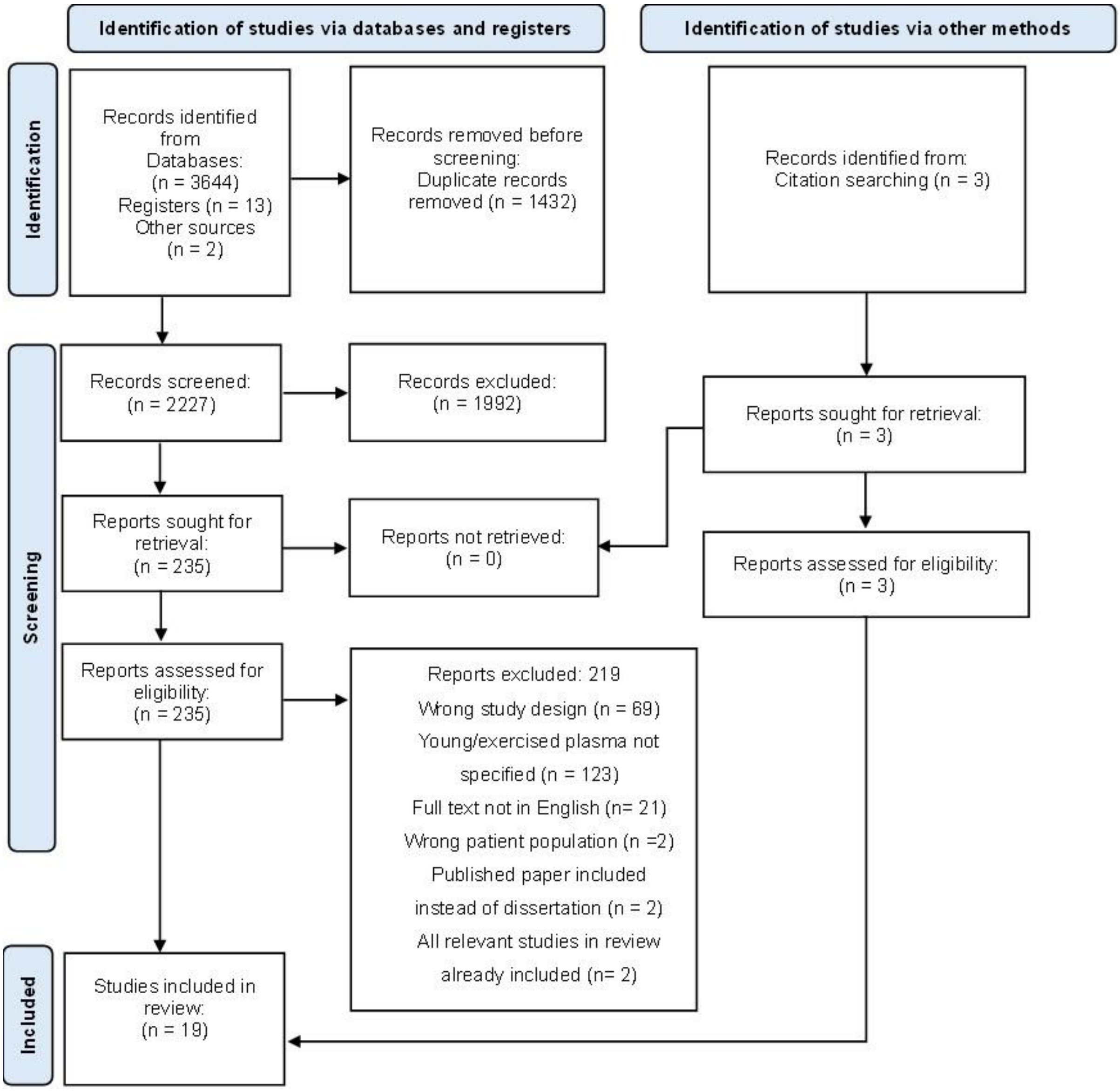

Search results and study selection and inclusion process [29].

SUPPLEMENTARY DATA

Appendix V

Supplementary Table 3- Quality assessment of included animal studies using SYRCLE's tool

STUDY	SELECTION BIAS			PERFORMANCE BIAS		DETECTION BIAS		ATTRITION BIAS	REPORTING BIAS	OTHER
	**Sequence generation**	**Baseline characteristics**	**Allocation concealment**	**Random housing**	**Blinding**	**Random outcome assessment**	**Blinding**	**Incomplete outcome data**	**Selective outcome reporting**	**Other sources of bias**
**Castello et al. [32]**	Unclear	Yes	Unclear	Unclear	Unclear	Yes	Yes	Unclear	Yes	No
**Hahn et al. [19]**	Unclear	Yes	Unclear	Unclear	No	No	No	Yes	Unclear	No
**Hernandez et al. [20]**	No	Yes	No	Unclear	No	No	No	No	Yes	No
**Schroer et al. [38]**	Yes	Yes	Yes	Unclear	Yes	Yes	Yes	Yes	Yes	Yes
**Smith et al. [36]**	Unclear	Yes	Unclear	Unclear	Yes	Unclear	Yes	Yes	Yes	Yes
**Xia et al. [33]**	Unclear	Yes	Unclear	Unclear	No	No	No	Yes	Yes	Yes
**Zhao et al. [25]**	No	Yes	No	Unclear	No	No	No	Yes	Yes	Yes
**Villeda et al. [34]**	Yes	Yes	Yes	Unclear	Yes	Yes	Yes	Yes	Yes	Yes
**Ghaffari-Nasab et al. [18]**	Unclear	Yes	Unclear	Unclear	No	Unclear	No	No	Yes	Yes
**Middeldorp et al. [21]**	No	Yes	No	Unclear	No	No	No	Unclear	Yes	Yes
**De Miguel et al. [37]**	Yes	Yes	Yes	Yes	Yes	No	Yes	Unclear	Yes	Yes
**Horowitz et al. [14]**	Unclear	Yes	Yes	Unclear	Yes	Unclear	Yes	Unclear	Yes	Yes
**Kim et al. [16]**	Unclear	Yes	Unclear	Unclear	No	Unclear	No	Yes	Yes	Yes
**Norevik et al. [17]**	Unclear	Yes	Yes	Unclear	Yes	Unclear	Yes	No	Yes	Yes
**Huuha et al. [15]**	Unclear	Yes	Yes	Unclear	Yes	Unclear	Yes	Yes	Yes	Yes
**Khrimian et al. [35]**	Unclear	Yes	Unclear	Unclear	Unclear	No	Yes	Unclear	Yes	Yes

© 2025. Kirton MJ et al. Published online at http://www.aginganddisease.org/EN/10.14336/AD.2025.0849

SUPPLEMENTARY DATA

Supplementary Figure 2- Risk of bias assessment using SYRCLE's risk of bias tool.

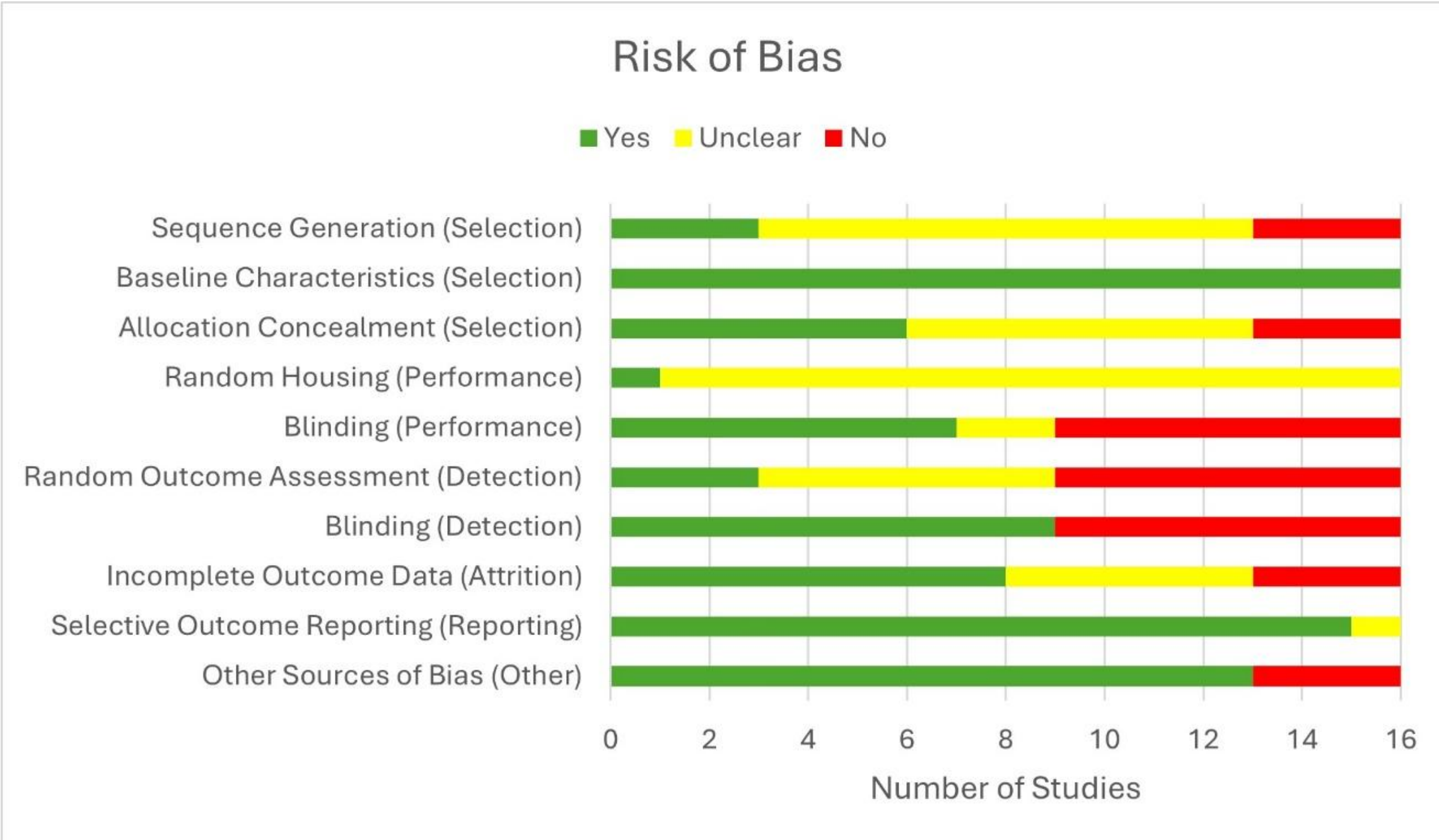

Supplementary Table 4- Quality assessment of included clinical studies using PEDro scale

References	Random allocation	Groups similar at BL	Concealed allocation	Assessor blinding	Subject blinding	Therapist blinding	BG statistical difference reported for PO	Point and variability measures reported	<15 % dropouts	Eligibility criteria specified	ITT analysis	Total PEDro score
**Parker et al. [23]**	**0**	**0**	**0**	**0**	**0**	**0**	**0**	**0**	**1**	**1**	**0**	**1**
**Sha et al. [24]**	**1**	**1**	**0**	**0**	**0**	**0**	**1**	**0**	**1**	**1**	**1**	**5**

© 2025. Kirton MJ et al. Published online at http://www.aginganddisease.org/EN/10.14336/AD.2025.0849
